# Disruption of functional network development in children with prenatal Zika virus exposure revealed by resting-state EEG

**DOI:** 10.1038/s41598-025-90860-0

**Published:** 2025-02-21

**Authors:** Ahmet Omurtag, Samah Abdulbaki, Thomas Thesen, Randall Waechter, Barbara Landon, Roberta Evans, Dennis Dlugos, Geetha Chari, A. Desiree LaBeaud, Yumna I. Hassan, Michelle Fernandes, Karen Blackmon

**Affiliations:** 1 Department of Engineering, Nottingham Trent University, Nottingham, UK; 2 Bio-Signal Group Inc., Acton, MA USA; 3 Geisel School of Medicine at Dartmouth and Dartmouth College, Hanover, NH USA; 4 Windward Islands Research and Education Foundation, St George’s University, St. George’s, West Indies Grenada; 5 St George’s University School of Medicine, St. George’s, West Indies Grenada; 6 Children’s Hospital of Philadelphia, Philadelphia, PA USA; 7 State University of New York Downstate Health Sciences University, New York, NY USA; 8 Department of Pediatrics, Stanford University School of Medicine, Stanford, CA USA; 9 National Health Service Clinical Scientist Training, Morriston Hospital, Swansea, UK; 10 Department of Paediatrics, University of Oxford, Oxford, UK; 11 Oxford Maternal and Perinatal Health Institute, Nuffield Department of Women’s and Reproductive Health, and Green Templeton College, University of Oxford, Oxford, UK; 12 Brain and Mind Institute, Aga Khan University, Nairobi, Kenya

**Keywords:** Neuroscience, Health care

## Abstract

Children born to mothers infected by Zika virus (ZIKV) during pregnancy are at increased risk of adverse neurodevelopmental outcomes including microcephaly, epilepsy, and neurocognitive deficits, collectively known as Congenital Zika Virus Syndrome. To study the impact of ZIKV on infant brain development, we collected resting-state electroencephalography (EEG) recordings from 28 normocephalic ZIKV-exposed children and 16 socio-demographically similar but unexposed children at 23–27 months of age. We assessed group differences in frequency band power and brain synchrony, as well as the relationship between these metrics and age. A significant difference (*p* < 0.05, Bonferroni corrected) in Inter-Site Phase Coherence was observed: median Pearson correlation coefficients were 0.15 in unexposed children and 0.07 in ZIKV-exposed children. Results showed that functional brain networks in the unexposed group were developing rapidly, in part by strengthening distal high-frequency and weakening proximal lower frequency connectivity, presumably reflecting normal synaptic growth, myelination and pruning. These maturation patterns were attenuated in the ZIKV-exposed group, suggesting that ZIKV exposure may contribute to neurodevelopmental vulnerabilities that can be detected and quantified by resting-state EEG.

**Supplementary Information:**

The online version contains supplementary material available at 10.1038/s41598-025-90860-0.

## Introduction

Congenital Zika virus (ZIKV) infection has been associated with severe neurological outcomes, such as microcephaly and epilepsy, in 4–6% of ZIKV-exposed children^1,2^. However, the long-term neurodevelopmental consequences of ZIKV exposure in normocephalic children remain poorly understood^3^. This knowledge gap hinders the development of targeted interventions and prognostic assessments for the 94–96% of ZIKV-exposed children who do not present with microcephaly at birth^4^.

Brain functional connectivity, which reflects the synchronization of neuronal activity across different regions, undergoes significant changes during early childhood^5^. These developmental processes are crucial for the establishment of cognitive and behavioural functions. Alterations in functional connectivity patterns have been linked to various neurodevelopmental disorders ^6^.

ZIKV has demonstrated neurotropism and the ability to disrupt neural progenitor cells, leading to abnormal brain development^7^ including disruptions to white matter organisation^8^. While structural brain abnormalities in ZIKV-exposed infants have been well-documented, the impact on functional connectivity remains largely unexplored^9^. Limited studies in animal models have suggested that ZIKV infection may alter neural network organization and synchronization^10,11^.

Electroencephalography (EEG) provides a non-invasive method to assess functional connectivity through the analysis of neural oscillations and their synchronization across brain regions^12^. In particular, inter-site phase clustering, a measure of phase synchronization between different EEG channels, has been used to quantify functional connectivity in various experimental and neurological conditions^13–16^.

Previous work by our team has reported subtle visual impairments in ZIKV-exposed normocephalic children, such as deficits in visual acuity and contrast sensitivity, despite the absence of other cognitive, motor, language, or behavioural delays^17^. These findings underscore the importance of developing functional methods to assess neural synchrony in normocephalic individuals who were prenatally exposed to the Zika virus. While structural imaging is invaluable for revealing physical abnormalities in the brain, it may not capture the subtle functional disruptions that can occur. This study aimed to address the current knowledge gap by investigating EEG-based functional connectivity in ZIKV-exposed normocephalic, nonepileptic children (ZEC) compared to demographically matched children unexposed to ZIKV (UC). By focusing on inter-site phase clustering (ISPC), we sought to elucidate potential alterations in brain network organization associated with prenatal ZIKV exposure in the absence of microcephaly or epilepsy, particularly in brain areas likely to be affected by the virus.

## Results

After pre-processing, EEG recording of 65 participants were retained as they matched the data quality criteria described in Methods. Analysis was further restricted to children aged 23–27 months to ensure balanced distribution of each group across the age window, as younger subjects were predominantly ZEC and older subjects UC. This age range also coincides with optimal neurodevelopmental assessment, representing the earliest period when: (i) neurodevelopment is not confounded by transient neurological syndromes of prematurity, and (ii) standard developmental instruments, like the Bayley Scales of Infant Development, show adequate medium- and long-term predictive validity^18^. Data from all 44 subjects within this age range were retained for analysis. The subpopulation selected for further analysis contained the groups UC (16, with 7 females) and ZEC (28, with 13 females), each group being nearly evenly distributed within the age-inclusion window. We used microEEG^19–21^, a portable, wireless EEG device, together with an analysis pipeline based on the identification of changes in ISPC with age. The average duration of EEG recordings in the analysis group was 12.8 min.

**Fig. 1:**
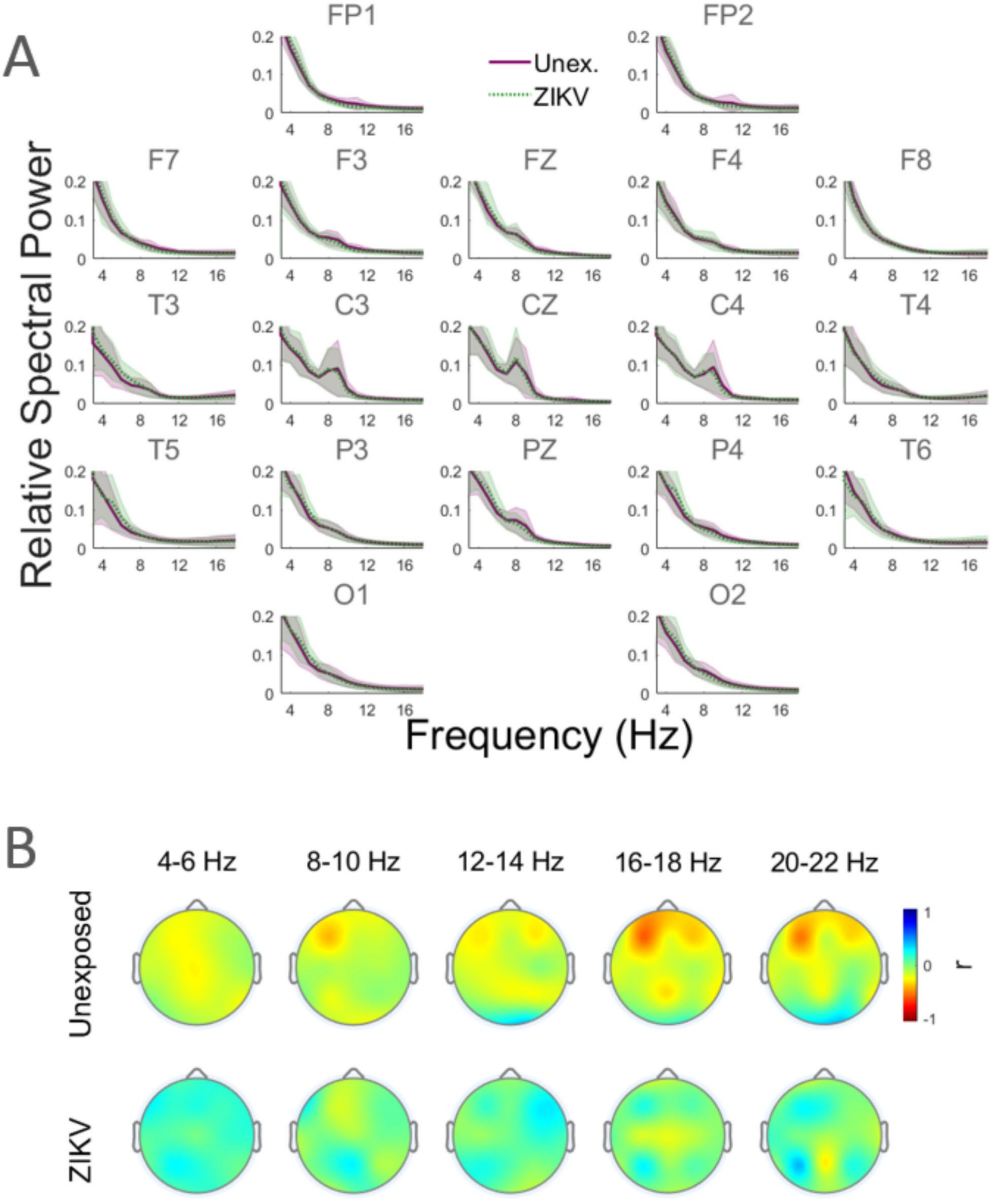
Relative band power and its age-dependence. (**A**) Relative frequency band power (FBP) as a function of frequency for each electrodes site. The UC (solid green line) and ZEC (dashed purple) averages are shown. Shaded regions are sample standard error. (**B**) Topographic illustration of the correlation of FBP with age for specific frequency ranges for the UC (top row) and ZEC (bottom).

The mean of Frequency Band Power (FBP) for the UC and ZEC groups are shown in Fig. 1A in subplots located approximately in the electrodes’ topographic locations. The plots show UC as the solid green lines and ZEC as dashed purple lines, with the corresponding shaded regions indicating the sample standard deviations. The figure shows prominent peaks in the spectra around 9 Hz in the central (C3, CZ, C4), nearby frontal (F3, FZ, F4) and central parietal (PZ) sites as well as a smaller frontopolar peak near 11 Hz. The small differences between UC and ZEC did not reach statistical significance for any of the frequency bands. Figure 1B shows the topographical distribution of the correlation of FBP with age averaged over specific representative frequency ranges, for UC (top row) and ZEC (bottom). The figure shows that the developmental decrease in UC which was small and widely distributed for 4–6 Hz progressively increased in magnitude with frequency and became focused on bilateral frontal electrodes at higher frequencies, with a left asymmetry particularly for frequencies > 16 Hz. These features were absent in ZEC which displayed overall small developmental increases. For > 12 Hz there was also small increasing trend for occipital FBP in UC. These trends in the frequency dependent correlations between FBP and age are further illustrated in Fig. S4 for each electrode. In addition, the patterns visually displayed in Fig. 1A are presented as band averaged values in Table 1.

**Table 1:** Band power at selected frequency ranges.

Electrode	4–6 Hz		6–8 Hz		8–10 Hz		22–24 Hz	
	UC	ZEC	UC	ZEC	UC	ZEC	UC	ZEC
FP1	0.144 ± 0.025	0.161 ± 0.035	0.063 ± 0.012	0.066 ± 0.017	0.036 ± 0.010	0.029 ± 0.008	0.012 ± 0.008	0.009 ± 0.007
FP2	0.139 ± 0.032	0.154 ± 0.035	0.061 ± 0.015	0.067 ± 0.019	0.034 ± 0.011	0.031 ± 0.009	0.013 ± 0.010	0.010 ± 0.008
F7	0.132 ± 0.036	0.144 ± 0.065	0.060 ± 0.011	0.064 ± 0.022	0.037 ± 0.009	0.033 ± 0.010	0.016 ± 0.009	0.013 ± 0.008
F3	0.125 ± 0.025	0.129 ± 0.042	0.067 ± 0.012	0.068 ± 0.019	0.049 ± 0.017	0.043 ± 0.016	0.014 ± 0.008	0.014 ± 0.009
FZ	0.156 ± 0.019	0.161 ± 0.031	0.081 ± 0.013	0.090 ± 0.027	0.054 ± 0.014	0.052 ± 0.023	0.006 ± 0.003	0.005 ± 0.003
F4	0.129 ± 0.030	0.123 ± 0.032	0.066 ± 0.012	0.069 ± 0.017	0.044 ± 0.008	0.046 ± 0.017	0.013 ± 0.009	0.014 ± 0.009
F8	0.136 ± 0.030	0.127 ± 0.032	0.061 ± 0.011	0.063 ± 0.016	0.036 ± 0.006	0.034 ± 0.008	0.013 ± 0.009	0.015 ± 0.008
T3	0.111 ± 0.049	0.124 ± 0.046	0.058 ± 0.023	0.073 ± 0.029	0.038 ± 0.017	0.042 ± 0.021	0.026 ± 0.018	0.018 ± 0.010
C3	0.128 ± 0.032	0.133 ± 0.038	0.084 ± 0.027	0.090 ± 0.033	0.073 ± 0.045	0.075 ± 0.044	0.008 ± 0.005	0.007 ± 0.003
CZ	0.150 ± 0.037	0.148 ± 0.038	0.093 ± 0.028	0.104 ± 0.041	0.089 ± 0.051	0.095 ± 0.060	0.003 ± 0.001	0.003 ± 0.001
C4	0.131 ± 0.031	0.132 ± 0.038	0.085 ± 0.024	0.089 ± 0.031	0.069 ± 0.037	0.071 ± 0.035	0.007 ± 0.003	0.008 ± 0.005
T4	0.119 ± 0.046	0.116 ± 0.041	0.059 ± 0.022	0.068 ± 0.027	0.036 ± 0.014	0.038 ± 0.017	0.022 ± 0.017	0.020 ± 0.013
T5	0.121 ± 0.065	0.121 ± 0.050	0.054 ± 0.022	0.070 ± 0.033	0.032 ± 0.012	0.032 ± 0.012	0.022 ± 0.018	0.019 ± 0.014
P3	0.148 ± 0.030	0.144 ± 0.028	0.073 ± 0.014	0.086 ± 0.025	0.047 ± 0.017	0.047 ± 0.017	0.008 ± 0.005	0.008 ± 0.004
PZ	0.153 ± 0.029	0.162 ± 0.030	0.087 ± 0.020	0.096 ± 0.034	0.063 ± 0.027	0.055 ± 0.022	0.005 ± 0.002	0.004 ± 0.002
P4	0.144 ± 0.033	0.148 ± 0.030	0.075 ± 0.014	0.084 ± 0.027	0.050 ± 0.016	0.043 ± 0.014	0.008 ± 0.004	0.008 ± 0.004
T6	0.128 ± 0.038	0.121 ± 0.047	0.063 ± 0.015	0.070 ± 0.033	0.035 ± 0.010	0.030 ± 0.009	0.018 ± 0.011	0.020 ± 0.013
O1	0.143 ± 0.054	0.148 ± 0.037	0.072 ± 0.020	0.086 ± 0.024	0.047 ± 0.020	0.045 ± 0.016	0.010 ± 0.011	0.008 ± 0.007
O2	0.140 ± 0.036	0.151 ± 0.031	0.078 ± 0.018	0.087 ± 0.024	0.053 ± 0.019	0.045 ± 0.015	0.008 ± 0.006	0.007 ± 0.005

The relative frequency band power for each electrode for specific frequency bands. The mean ± standard error values are shown.

**Fig. 2:**
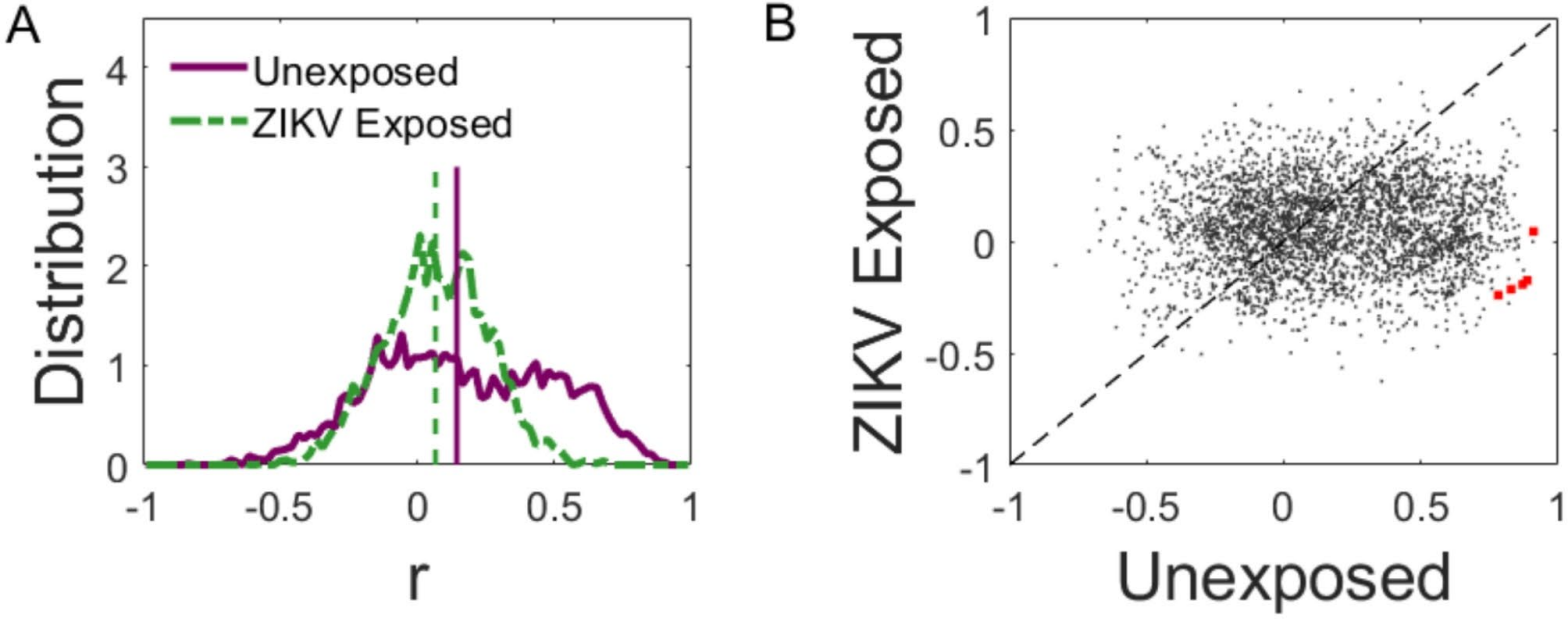
Distribution of developmental change in synchrony. (**A**) Histograms of *r*, the correlation of ISPC with age in the UC (purple) and ZEC (green). Corresponding medians (0.15 and 0.07, respectively) are shown by the dashed vertical lines; (**B**) Scatter plot of *r*_*N*_* and r*_*Z*_, with each dot corresponding to an electrode pair and specific frequency, and the diagonal dashed line showing *r*_*N*_ = *r*_*Z*_.

Next, we investigated developmental patterns in functional synchrony using ISPC. The histograms of ISPC development for UC and ZEC are shown in Fig. 2. We use the term ISPC development to describe the correlation between ISPC and age, denoted *r*_*N*_ and *r*_*Z*_, for UC and ZEC respectively. The histogram in UC (purple curve in Fig. 2A) appears bimodal with one cluster of connections centred around *r*_*N*_ = 0. The left tail in the distribution of this cluster is thicker than the left tail in the distribution of *r*_*Z*_ (green curve), suggesting that developmental weakening of synchrony in UC was more extensive than in ZEC. Another cluster in the distribution of *r*_*N*_ peaks around *r*_*N*_ = 0.5 and extends in the positive direction beyond the right tail of the distribution of *r*_*Z*_, indicating that ISPC developmental increases were stronger in UC than in ZEC. The medians of the two populations, shown by vertical dashed lines, were both positive. Both groups’ ISPC values increased with age; however, the UC’s median (0.15) was greater than that of ZEC (0.07), although not reaching statistical significance. ISPC values are provided in Table 2.

**Table 2:** Connectivity and its development in the UC and ZEC groups.

Zone index	Sites	Freq. band (Hz)	Cortical distance (mm)	ISPC						Correlation of ISPC with age	
				Mean		Median		Standard Error			
				UC	ZEC	UC	ZEC	UC	ZEC	UC	ZEC
1	FP2–P3	24–26	157	0.122	0.115	0.122	0.115	0.018	0.007	0.807	0.317
	FP2–O1	24–26	175	0.157	0.143	0.157	0.143	0.051	0.022	0.577	− 0.055
	FP2–T5	24–26	160	0.118	0.113	0.118	0.113	0.015	0.005	0.509	0.146
	FP1–P4	24–26	156	0.113	0.113	0.113	0.113	0.007	0.008	0.656	− 0.362
	FP1–O1	24–26	166	0.14	0.128	0.14	0.128	0.026	0.011	0.544	− 0.03
	FP1–O2	24–26	173	0.133	0.123	0.133	0.123	0.026	0.014	0.605	− 0.064
	FP1–T6	24–26	159	0.115	0.114	0.115	0.114	0.012	0.009	0.515	− 0.115
	F4–O1	24–26	156	0.132	0.122	0.132	0.122	0.024	0.011	0.753	− 0.004
2	FP1–T4	24–26	132	0.116	0.115	0.116	0.115	0.011	0.007	0.535	− 0.072
	F4–P3	24–26	132	0.116	0.112	0.116	0.112	0.009	0.006	0.761	0.174
	F3–P4	24–26	131	0.113	0.113	0.113	0.113	0.01	0.006	0.731	− 0.026
	C3–F8	24–26	132	0.114	0.116	0.114	0.116	0.008	0.008	0.515	− 0.257
	C3–T6	24–26	131	0.116	0.113	0.116	0.113	0.008	0.007	0.764	0.278
	FZ–T6	24–26	132	0.117	0.115	0.117	0.115	0.012	0.01	0.726	0.235
	O2–F8	24–26	133	0.151	0.141	0.151	0.141	0.031	0.019	0.624	0
3	F4–PZ	24–26	113	0.123	0.12	0.123	0.12	0.011	0.007	0.506	− 0.051
	P4–FZ	24–26	112	0.117	0.116	0.117	0.116	0.008	0.007	0.762	0.124
	P4–F8	24–26	110	0.127	0.118	0.127	0.118	0.022	0.008	0.506	0.201
	P3–FZ	24–26	113	0.12	0.116	0.12	0.116	0.012	0.009	0.8	0.328
	P3–T6	24–26	110	0.121	0.121	0.121	0.121	0.016	0.013	0.728	0.285
4	FP2–F7	24–26	90	0.122	0.121	0.122	0.121	0.015	0.011	0.84	0.061
	P4–O1	24–26	86	0.152	0.138	0.152	0.138	0.044	0.018	0.646	− 0.069
	P3–O2	24–26	83	0.149	0.134	0.149	0.134	0.046	0.035	0.658	0.246
	O1–T6	24–26	90	0.147	0.14	0.147	0.14	0.04	0.029	0.66	− 0.2
5	FP2–P3	20–22	157	0.125	0.12	0.125	0.12	0.014	0.008	0.821	0.545
	FP1–P4	20–22	156	0.121	0.118	0.121	0.118	0.008	0.007	0.705	− 0.185
	F4–O1	20–22	156	0.131	0.124	0.131	0.124	0.015	0.008	0.701	0.141
	F3–O2	20–22	152	0.123	0.123	0.123	0.123	0.008	0.005	0.626	0.303
	O1–F8	20–22	154	0.147	0.135	0.147	0.135	0.026	0.013	0.677	0.264
	F7–O2	20–22	153	0.127	0.124	0.127	0.124	0.014	0.006	0.511	0.246
6	P4–FZ	20–22	112	0.121	0.121	0.121	0.121	0.007	0.008	0.73	0.293
	P3–FZ	20–22	113	0.127	0.119	0.127	0.119	0.013	0.007	0.7	− 0.016
	P3–F7	20–22	111	0.123	0.121	0.123	0.121	0.01	0.007	0.504	0.366
7	P4–O1	18–20	86	0.16	0.148	0.16	0.148	0.036	0.02	0.639	− 0.012
	O1–T6	18–20	90	0.16	0.157	0.16	0.157	0.045	0.037	0.548	− 0.061
8	P4–O1	4–6	86	0.278	0.294	0.278	0.294	0.035	0.062	− 0.482	− 0.049
	O1–T6	4–6	90	0.258	0.262	0.258	0.262	0.032	0.037	− 0.653	0.074
	PZ–T5	4–6	87	0.284	0.299	0.284	0.299	0.037	0.045	− 0.512	0.088
	PZ–T6	4–6	85	0.296	0.305	0.296	0.305	0.037	0.046	− 0.71	− 0.044
	O2–T5	4–6	88	0.255	0.266	0.255	0.266	0.029	0.046	− 0.659	0.088
9	C4–O1	8–10	123	0.193	0.189	0.193	0.189	0.017	0.013	− 0.31	− 0.217
	C3–O2	8–10	119	0.194	0.191	0.194	0.191	0.011	0.014	− 0.502	− 0.116
	O1–T4	8–10	124	0.18	0.174	0.18	0.174	0.012	0.01	− 0.37	− 0.486
	O2–T3	8–10	122	0.178	0.176	0.178	0.176	0.009	0.011	− 0.677	0.275
10	F4–T5	6–8	151	0.19	0.194	0.19	0.194	0.008	0.009	0.463	− 0.237
	F3–O2	6–8	152	0.198	0.198	0.198	0.198	0.016	0.008	0.17	− 0.188
	O1–F8	6–8	154	0.209	0.207	0.209	0.207	0.015	0.013	− 0.538	− 0.193
	F7–O2	6–8	153	0.206	0.203	0.206	0.203	0.013	0.012	− 0.044	− 0.074
	F7–T6	6–8	152	0.192	0.194	0.192	0.194	0.009	0.007	0.471	− 0.48
	F8–T5	6–8	153	0.195	0.195	0.195	0.195	0.009	0.01	− 0.04	− 0.243

Selected connections are shown from the numbered regions in Fig. 7 (column titled Index). Cortical distance based on MNI coordinates of the electrodes on adult cortex.

Group differences are further highlighted by Fig. 2B, where each dot represents a pair of values *r*_*N*_ and *r*_*Z*_ for one connection, i.e. the developmental change of ISPC in the UC (x-axis) and ZEC (y-axis). Had the development of connectivity been unaffected by ZIKV exposure, the dots would be symmetrically arranged around the dotted diagonal line. The observed distribution, however, is skewed toward the right. Many electrode pairs that show ISPC increases with age in UC (centred approximately at *r*_*N*_ = 0.5) appear to be mainly stagnant in ZEC (centred around *r*_*Z*_ = 0). The red dots in Fig. 2B show the electrode pairs in which ISPC development was significantly higher in UC than in ZEC (*p* < 0.05, Bonferroni corrected). These correspond to ISPC in the infant gamma frequency range, specifically, phase clustering between the left inferior frontal scalp area and the right frontopolar (FP2-F7 at 28–38 Hz) and right parietal areas (P4-F7 at 28–30 Hz).

**Fig. 3:**
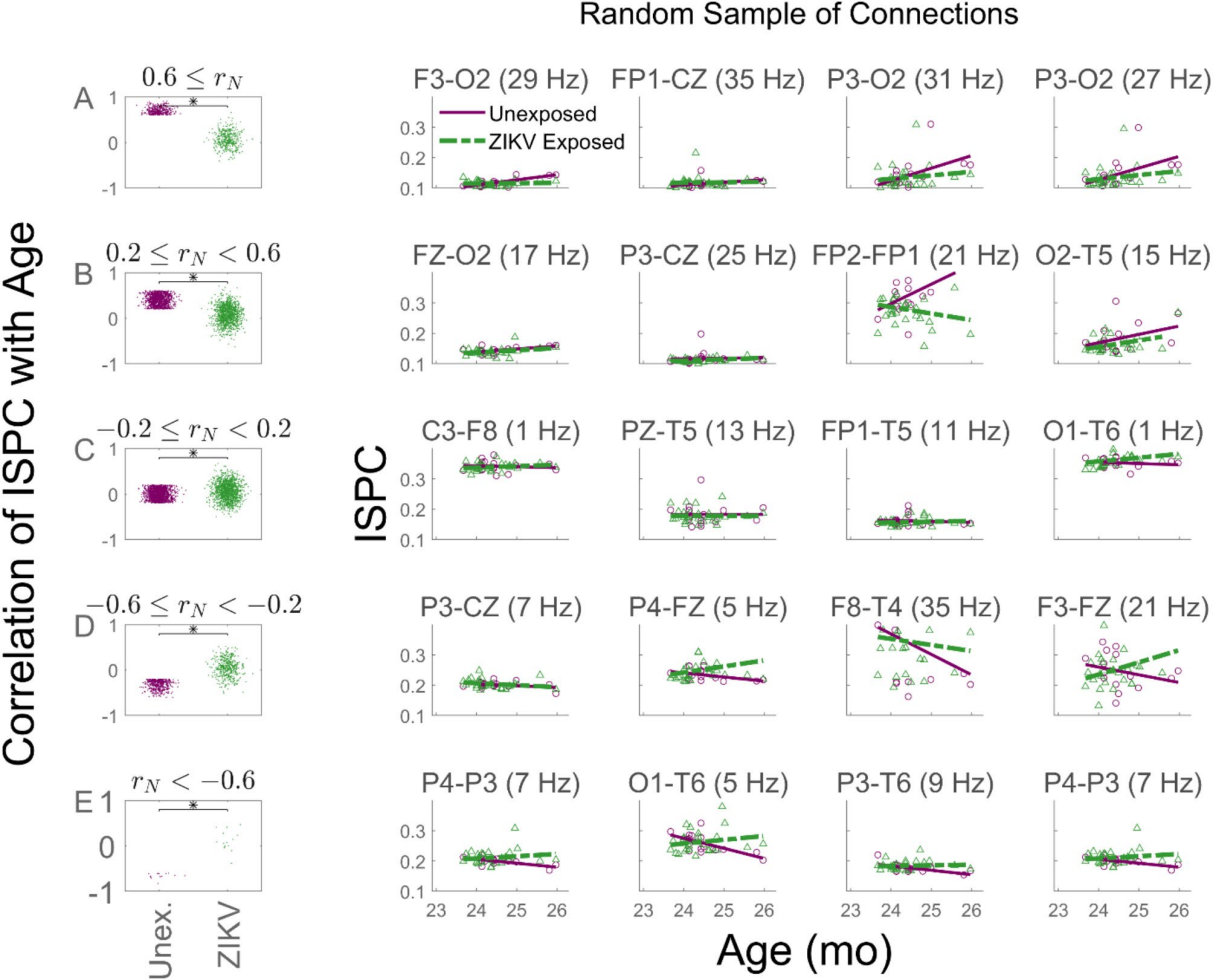
Developmental change in synchrony. The correlation strength between inter-site phase clustering (ISPC) and age in UC and ZEC are grouped into rows depending on their effect size in UC. (**A**) Rapidly increasing; (**B**) moderately increasing; (**C**) stagnating; (**D**) moderately decreasing; (**E**) rapidly decreasing. Columns 2–5 show randomly selected examples of electrode pairs, separated into UC (purple circles) and ZEC (green triangles) groups, together with their best-fit lines.

To investigate developmental differences in phase clustering across specific sites, we segregated ISPC development values in Fig. 3 according to their speed of development in UC. They were divided into the groups (1) rapidly increasing (top row), 0.6 < *r*_*N*_; (2) moderately increasing (second row), 0.2 ≤ *r*_*N*_ < 0.6; (3) stagnating (third row), − 0.2 ≤ *r*_*N*_ < 0.2; (4) moderately decreasing (fourth row), − 0.6 ≤ *r*_*N*_ < –0.2; and (5) rapidly decreasing (bottom row), *r*_*N*_ < − 0.6. In the leftmost column, the ISPC’s correlation with age in UC (purple dots) is compared with the corresponding values in ZEC (green) for the same electrode pairs. The left coloumn of the figure indicates that the ISPC developmental increases in UC (positive *r*_*N*_) outnumbered decreases (negative *r*_*N*_), and in each case the developmental changes of the same electrode pairs in ZEC were much smaller. This is illustrated by the fact that the cluster of green dots was located closer to zero than the cluster of purple dots. The columns 2–5 of Fig. 3 display examples of ISPC developmental changes with age for specific pairs randomly selected from the full set of electrode pairs.

**Fig. 4:**
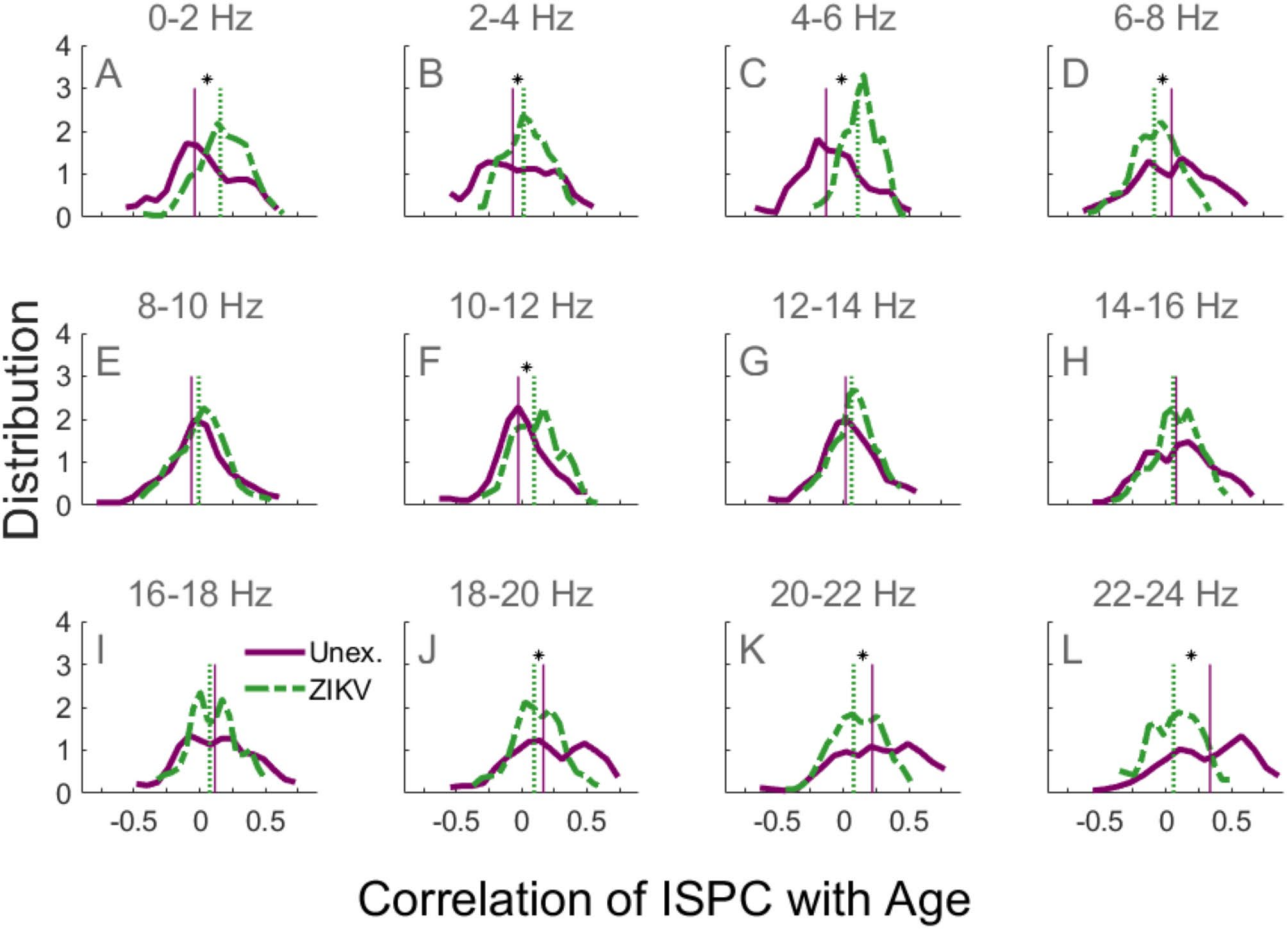
The distribution of the developmental change in synchrony in frequency bands. UC (solid purple) and ZEC (green dashed) for the frequency bands indicated above each subplot. Plots show histograms of *r*, the correlation between ISPC and age. The vertical lines indicate the medians of the corresponding distributions. Distributions whose medians differ significantly are indicated using an asterisk located between the vertical lines showing the medians (*p < 0.05, Bonferroni corrected).

Figure 2 provides a developmental outline without indicating the detailed comparative changes in specific ISPC development. Figure 3 segregates ISPC according to their developmental speed. Figure 4 segregates connections by frequency band and suggests that UC and ZEC connectivity changes are highly frequency specific. There was a salient tendency for low frequency (0–6 Hz) ISPC values to weaken with age in UC while stagnating or strengthening in ZEC (Fig. 4A–C). The figure also showed that in 6–8 Hz, UC had significantly faster ISPC development than ZEC (Fig. 4D). A more striking shift occurred in the high frequency bands where ISPC in UC tended to strengthen with age (indicated by the right tail of the purple curves) while this was much more attenuated in ZEC (Fig. 4J–L).

**Fig. 5:**
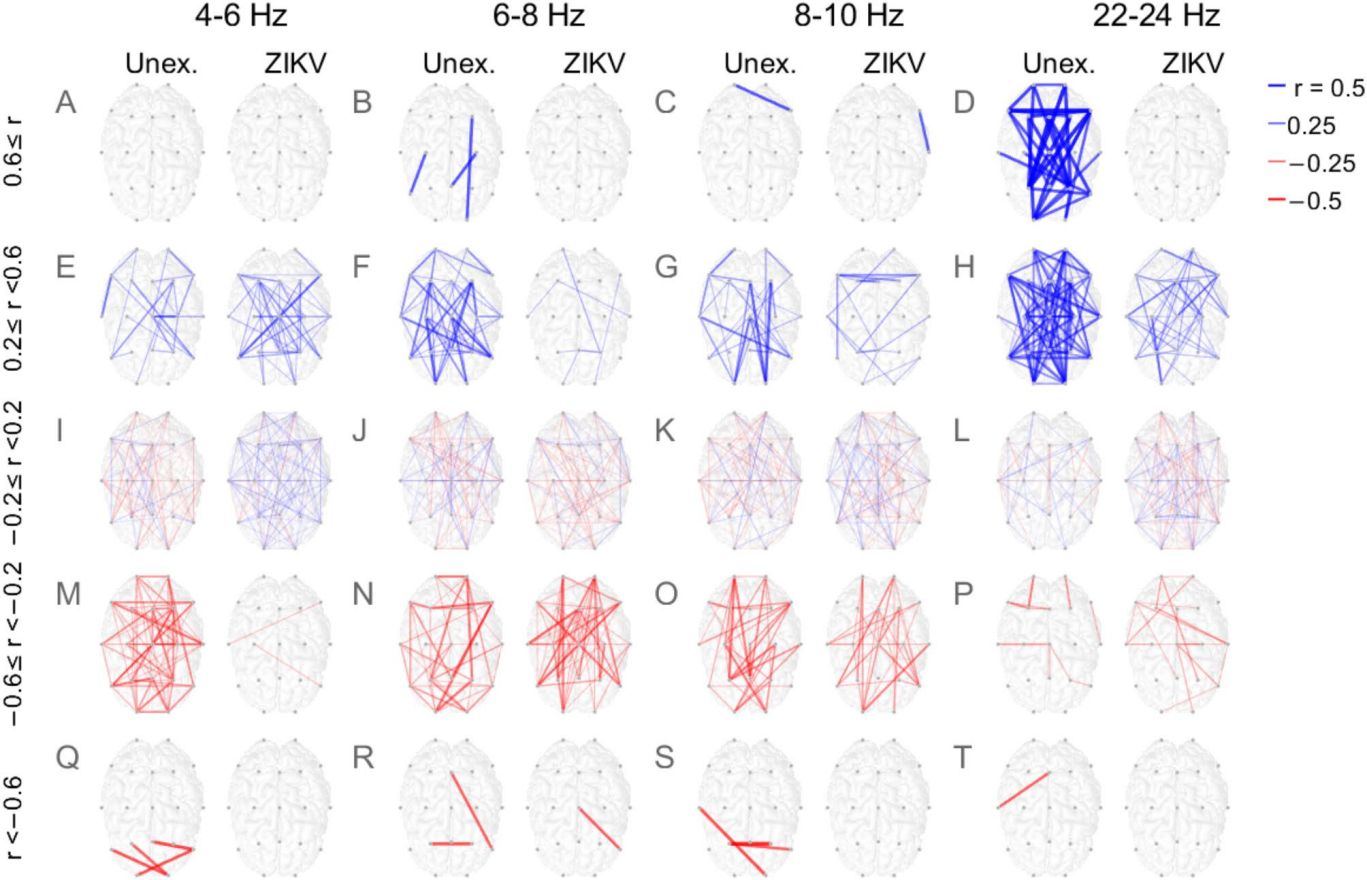
Topographic illustration of the developmental changes in connectivity. The frequency band for each pair of columns is shown at the top. Within each pair of columns, UC (left) and ZEC (right) connectivity development are shown as lines. Each row corresponds to a different development speed. (**A**–**D**) Rapidly developing; (**E**–**H**) moderately developing; (**I**–**L**) stagnating; (**M**–**P**) moderately weakening; (**Q**–**T**) rapidly weakening. A line’s colour indicates the sign of the change (blue/red for increase/decrease in ISPC with age) and the line’s thickness is proportional to the magnitude of the change, as indicated in the figure legend at top right.

In addition, we investigated the topographic distribution of specific electrode pairs. Figure 5 illustrates all connections that were clustered in ranges of frequency (columns) and of developmental speed (rows). Due to space constraints only a subset of frequency bands are shown and these bands were selected as follows: Using Fig. 4, two adjacent bands that differed sharply between ZEC and UC, and where median normal connectivity was weakening (4–6 Hz) and strengthening (6–8 Hz) were selected. In addition, 8–12 Hz, where the two groups’ developments were similar, both mainly stagnating, was selected. We also chose 22–24 Hz to exemplify rapidly developing EUC connectivity that contrasted with stagnation in ZEC.

Figure 5M,Q indicate extensive weakening of low-frequency connections in UC, which was almost completely absent from ZEC. Figure 5Q shows rapid weakening of UC’s occipitotemporal mainly interhemispheric connections at 4–6 Hz, while Fig. 5M indicates pervasive intra-hemispheric weakening. Furthermore, Fig. 5B,F show the development of a network linking occipital areas with central and frontal, and parietal with frontal areas at 6–8 Hz in UC. This included the normal increased development of intra-hemispheric long-distance connections. The figure indicates that these developmental changes were largely absent in ZEC. The high frequency (22–24 Hz) connections shown in Fig. 5D,H suggest the presence of normal development of networks that tightly link occipital, central and frontal areas both inter- and intra-hemispherically. Similar patterns of rapid normal development of pervasive high frequency networks were observed also in higher frequency bands (not included in this figure).

**Fig. 6:**
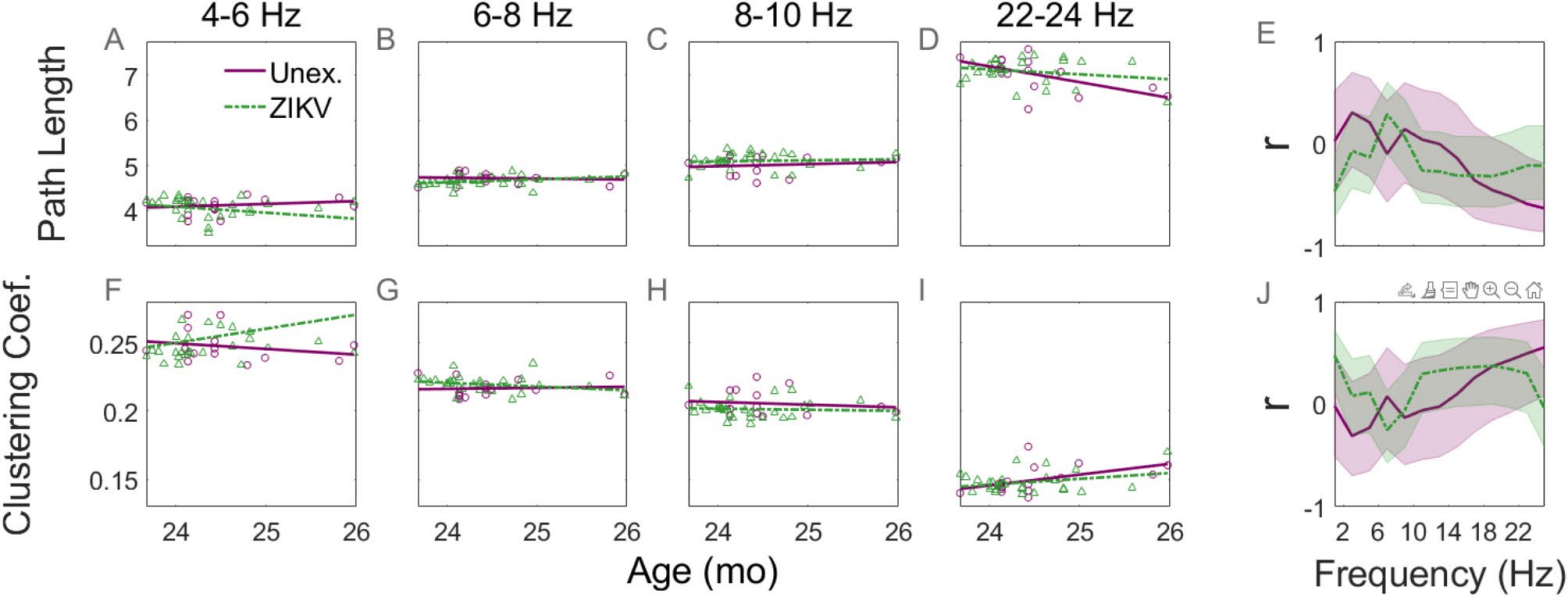
Graph theoretic characterisation of networks and their dependence on frequency. The characteristic path length (**A**–**D**) and the clustering coefficient (**F**–**I**), based on all pairs of channels at all frequency bands, in UC (purple solid) and ZEC (green dashed). The values for individual UC (circles) and ZEC (triangles) and their best fit lines are shown. The rightmost column shows the correlation of the path length (**E**) and of the clustering coefficient (**J**) with age, as a function of the frequency of the connection. The shaded regions around each curve indicate the sample standard deviation.

Having examined the topographic distribution of connections, we next investigated graph theoretic measures based on ISPC. Figure 6 shows, for specific frequency bands, the values of the characteristic path length (A–D) and clustering coefficient (F–I) for UC (purple open circles) and ZEC (green triangles) as a function of the children’s age. In the rightmost column (E and J), developmental network changes are shown as a function of the frequency in the horizontal axis. These indicate that for networks with > 18 Hz the characteristic path lengths of the UC networks were decreasing while their clustering coefficients were increasing with age (purple solid curves), suggesting a progression toward small-world properties. In the low frequency range (~ 4 Hz) of the same graphs a developmental tendency in the opposite direction was evident. Such patterns were not found in ZEC (green dashed).

**Fig. 7:**
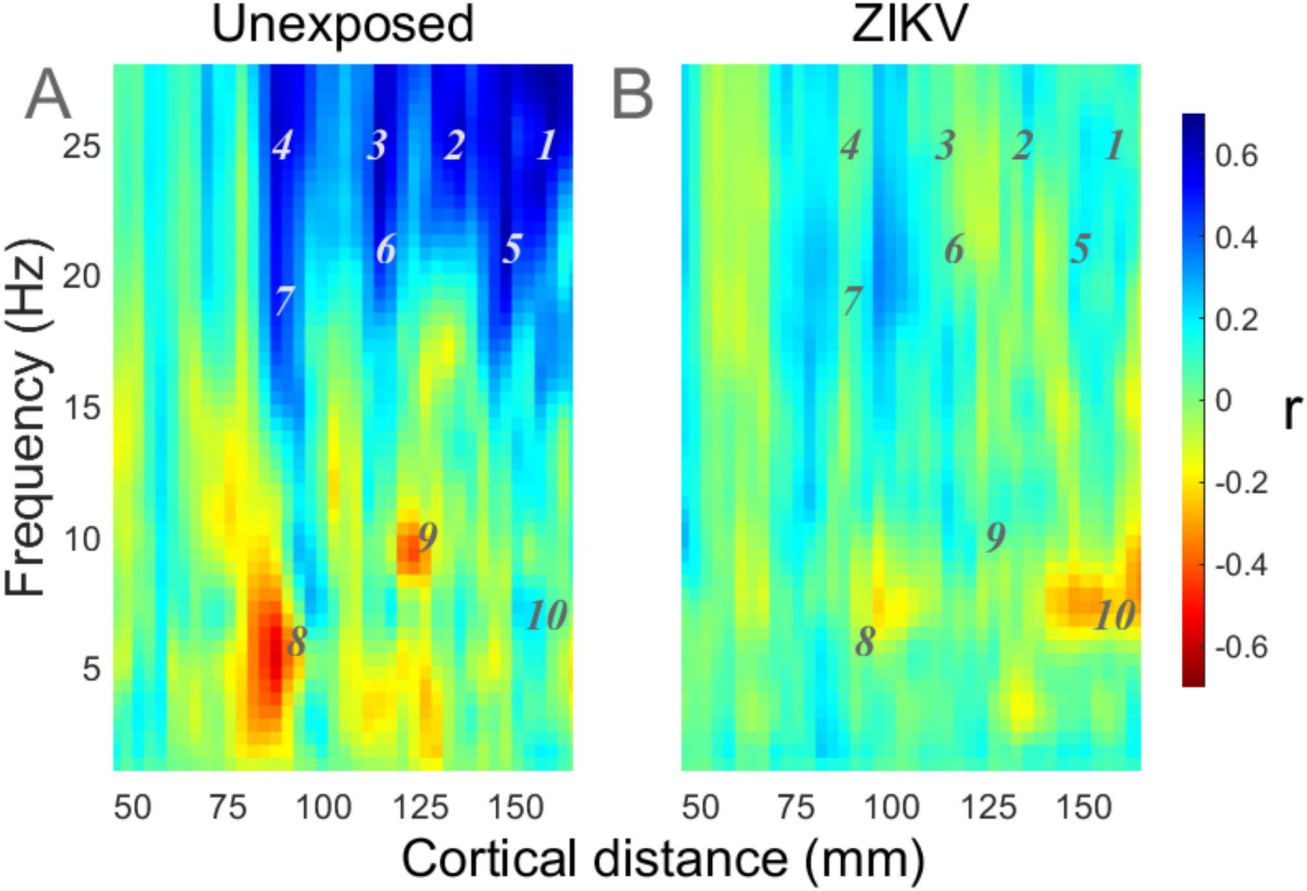
Dependence of connectivity development on frequency and cortical distance. Distances based on the MNI locations of primary neuronal populations in adult cortex associated with each electrode, for the UC (**A**) and ZEC (**B**). See Table 2 for selected electrode pairs and their values of ISPC at the numbered zones 1–10.

We have also investigated whether the developmental patterns studied above had a systematic dependence on the connections’ distances. Figure 7 plots *r*_*N*_ and *r*_*Z*_ as a function of the cortical distance (x-axis) and frequency (y-axis). The cortical distances were computed based on the Montreal Neurological Institute (MNI) coordinates of the main neuronal populations associated with each electrode ^22^. For this purpose, we have used the distances for the adult cortex since no equivalent study was available for our participants’ age group and for this study only the relative differences were of interest. To plot Fig. 7A the values of *r*_*N*_ were first linearly interpolated over a two-dimensional grid of cortical distance (with a resolution of 3 mm) versus frequency (resolution 0.5 Hz) then smoothed by a 3 × 3 convolution matrix. A similar procedure was followed for Fig. 7B and *r*_*Z*_. The vertical streaks in the figure follow from the fact that due to the arrangement of the 10–20 placement system many connections with a range of frequencies share the same approximate cortical distance. Figure 7A shows that high frequency connections were rapidly increasing with age in UC particularly for the medium and long-range connections (blue areas labelled 1–7). In addition, smaller groups of connections (zones 8 and 9) were being weakened in UC. Similar developmental changes were not visible in Fig. 7B (ZEC). Figure 7 reveals that long range connections were the primary cause of UC having a significantly greater number of connections getting stronger with age (Fig. 4J–L that for > 18 Hz). Table 2 partly lists the pairs of electrodes that gave rise to the patterns in Fig. 7. Only the connections in the zones labelled 1–10 are listed in the table. It suggests that the increasing connectivity in zones 1–7 included links between frontopolar and occipital, temporal and parietal areas of the brain.

## Discussion

We investigated developmental patterns in brain synchrony of children 23–27 months of age and how they differed with age between ZEC and UC. Local synchronisation based on frequency band power (Fig. 1) and graph theoretic network properties based on ISPC (Fig. 6) were also investigated. There were meaningful changes in brain synchrony associated with age in the UC that were not present in ZEC. Specifically, age-associated regional increases and decreases in brain synchrony were observed in UC that were not present in ZEC. This depended on frequency (Fig. 4), topography (Fig. 5) as well as cortical distance (Fig. 7). These results showed that UC undergo extensive developmental strengthening (particularly in the long range, high frequency connections) as well as some weakening of functional connections, and their networks at higher frequencies were trending toward small-world properties (Fig. 6). Importantly, these patterns appeared to be attenuated or even partly reversed in exposed children. These findings should be considered in the context of our prior report of ZEC from this cohort exhibiting subtle yet significant visual impairments, including reduced visual acuity and contrast sensitivity, despite otherwise typical development at 2 years of age ^17^. This suggests a potential association between altered ISPC and the observed visual dysfunction.

Given the critical role of neural synchronization in visual perception, as emphasized by previous findings ^23^, it is plausible that disruptions in ISPC during fetal development may contribute to the visual deficits identified in our ZEC cohort. While direct research on ISPC and prenatal visual development is limited, the broader concept of functional connectivity, as previously outlined^24^, offers a theoretical framework to explore how aberrant neural network organization might underlie these visual impairments. Further investigation into this relationship is warranted to elucidate the pathophysiological mechanisms underlying ZIKV-associated visual dysfunction.

The functional neurophysiology of normally developing infants and children has been characterized in previous studies^25–31^. Studies of local synchronisation found that a peak in the power spectrum resembling the adult alpha peak emerges around 3 months of age near 3–5 Hz, which shifts to higher frequencies with time. At the end of the first year, it is at 6–7 Hz and at 2 years around 8–9 Hz^28,30^. It has long been known that the mu-rhythm at central scalp locations has a similar early developmental pattern^32,33^. Generally, the boundaries of the typical frequency bands are lower in infants and children than in adults^34,35^. Given the ages of the subjects in our study (23–27 mo) the spectra in Fig. 1A with a local peak around 8–9 Hz in the central electrodes C3, CZ, C4 and PZ were consistent with such results. In adults the central mu-rhythm appearing over sensorimotor areas is attenuated by actual and intended movement, hence the appearance of this peak likely correlates with the development of motor and locomotor skills. The top row of Fig. 1B showed distributed small developmental decreases in local synchronisation in the unexposed children (more frontally focused at frequencies > 16 Hz).

EEG spectra may shift due to progressive myelination, changes in the orientation and density of neuronal assemblies and maturational alteration in skull and supportive tissue ^36^. During childhood there is a general reduction in the amplitude of neural oscillations over a wide frequency range^37^. The distributed decrease at low frequencies that we have found resembled the lack of topographic specificity in the age-related reduction of low frequency signals in adolescents^30,38^. This may be a long-term trend linked to developmental synaptic pruning and decreases in cortical gray matter density ^39^ as well as metabolic rate ^40^. Results in Fig. 1B also suggest that the developmental trends in frequency band power could discriminate between UC and ZEC. In a previous study, the difference in the change of resting state EEG power leading up to 24 months of age were found to discriminate between infants at low and high risk of Autism Spectrum Disorder (ASD)^41^.

Our findings suggest that functional connections in normally developing children undergo vigorous changes, with some connections being strengthened and others (a smaller number) being weakened. This was evidenced by the fact that in Fig. 2 the histogram for UC had higher median and was broader (both the positive and negative tails) than that of ZEC. The frequency specific histograms in Fig. 4 further suggested that weakening connections in UC were largely in the range 4–6 Hz while the strengthening ones were in 6–8 Hz and ≥ 14 Hz. These may be early manifestations of a longer-term trend (particularly Fig. 4D, H–L) as from 6 years old until early adolescence neural phase synchrony in the theta, beta and gamma bands are known to increase, accompanied by performance improvements in the detection and successful grouping of stimuli into a coherent precept during perception tasks^42^.

Analysis of the age-dependence of ISPC revealed systematic differences between UC and ZEC, despite the absence of significant group differences in ISPC values themselves (Figs. 4, 5 and 7, Table 2). Similarly, while neurodevelopmental test results showed no significant group differences between UC and ZEC, the age-dependent correlation with visual acuity scores (Cardiff Visual Acuity LogMar scores) differed significantly between groups (Figs S1 and S2). Notably, the visual deficits that were apparent at 2 years of age in our cohort^17^ had resolved by school age^43^, which speaks to the plasticity of the developing brain^44^. It is worth noting that both UC and ZEC were later exposed to a Responsive Caregiving Intervention after the EEG data for the current study were collected^44^. This could have contributed to later neurodevelopmental gain and reiterates the importance of directing at-risk children to appropriate early intervention programs.

We found that the dynamics of normal development shows a marked tendency toward small-world properties at higher frequencies (Fig. 6E,J), as a result of the downward trend in the characteristic path length (Fig. 6D) and the upward trend in the clustering coefficient (Fig. 6I). These suggest a shift towards random and lattice-like network characteristics, respectively, consistent with an increase in Small-World Propensity^45^. This partly agrees with the results of previous resting-state EEG studies of children^26,46^. MEG recordings of infants 0–60 months of age during rest and a prehension task found that mu-rhythm based sensorimotor networks progressively shifted toward small-world architecture after 1 year of age^47^. Small-world architecture is ubiquitous in functional brain networks^48,49^, has been found in structural brain networks of neonates^50^ and even cultured neuronal populations^51^. It supports both high segregation and integration ^52^ and its underdevelopment has been implicated in developmental deficiencies, aging and neurological disorders^53–57^. Children with Autism Spectrum Disorder (who share co-morbidities with ZEC) exhibited significantly lower clustering coefficient and higher characteristic path length than controls at 2 years of age^58^.

Early childhood is a period when fundamental skills—language, motor, cognition, memory—mature rapidly in ways that predict future functioning, and patterns of EEG development accompany these trends. Functional Magnetic Resonance Imaging results have shown that early changes occur in frontal and parietal activations that attend improvements in memory, executive control and visual processing^59–62^. These may be underpinned by the development of white matter (that continues until early adulthood) which contributes to the maturation of long-range synchronisation between cortical regions by increasing the speed and precision of propagation ^63,64^. This is consistent with the rapid increases in distal high-frequency connections we found in UC (Fig. 7A), since BOLD amplitude is tightly linked to gamma synchrony^65^. It would have been valuable to collect EEG at later timepoints in our cohort to determine whether ZEC show similar maturational gains on EEG metrics that correspond to the developmental gains observed following their participation in a Responsive Caregiving intervention^44^ and observed clinically at 3 and 4 years of age^43^.

The limitations of this study include the following. We have studied session averaged ISPC whereas the time variability of functional connectivity may carry additional clinically useful information^66^. The phase difference between signals can be examined as an additional metric associated with development, providing a finer discrimination capability that ISPC lacks, as it indiscriminately pools phase clustering without considering phase differences. The evolution of phase lags in experimental conditions have been reported^67^ but their clinical significance remains to be studied. We acknowledge the study’s limited sample size, which may impact generalisability and precluded analysis of sex differences and the degree to which functional connectivity mediates the relationship between ZIKV exposure and neurodevelopmental outcomes, particularly visual deficits. Finally, we have used 19 electrodes to calculate sensor level connectivity whereas a higher density coverage of electrodes on the scalp allowing the extraction of source activity may reveal additional developmentally and clinically relevant information^68^.

Recent surveillance data from the Centers for Disease Control and World Health Organization indicate that ZIKV transmission rates have decreased significantly compared to the 2015–2016 outbreak. However, sporadic cases continue to be reported in endemic regions as ZIKV continues to circulate globally particularly in tropical and subtropical regions^69–71^. Through the analysis of resting-state measurements performed with a portable EEG system, we have uncovered evidence that prenatal ZIKV exposure disrupts the development of large-scale neural network synchrony, as measured at 2 years of age. The extent to which this is transient and recoverable remains unknown. Further, the functional implications for the disrupted large-scale neural network development now or in the future remain unknown. Our findings shed light on the disruption of normal changes in signal amplitude and shifts from proximal to largely distal functional connectivity, as well as network trends toward small-world properties in young children. These could form the basis for developing practical methods of EEG scanning to assess neurodevelopment, particularly valuable for nonverbal populations, for the purpose of early and targeted provision of therapeutic resources. This approach could be especially beneficial for addressing global child health disparities, particularly in resource-limited settings where children may be disproportionately vulnerable to teratogenic exposures, preterm birth, and malnutrition. By focusing on normocephalic children, who may otherwise be overlooked, this study suggests that it is feasible to use cost-effective portable EEG with integrated advanced analysis to study and manage the spectrum of ZIKV’s neurodevelopmental effects.

## Methods

### Participants and socio-demographic and developmental metrics

This study used data from a study where children were recruited from a cohort of mother–child dyads who completed extensive serological and sociodemographic characterization during the 2016–2017 ZIKV outbreak in Grenada, West Indies, following routine antenatal appointments. Neurodevelopmental, visual acuity, and contrast sensitivity assessments, including cognition, motor and language skills, and behaviour, were clinically evaluated using the International Neurodevelopmental Assessment (INTER-NDA)^72^. Detailed description of the participant population, assessment procedures and results are summarised in Tables S1 and S2 in Supplementary Information. The study was approved by the Institutional Review Boards of St. George’s University, Grenada, West Indies (IRB#16061) and Stanford University, USA (IRB#s 37004 and 45242), and granted research clearance by the Grenada Ministry of Health. Mothers provided written informed consent for themselves and on behalf of their participating children and all methods were carried out in accordance with relevant guidelines and regulations.

### Data collection

The microEEG® 19-channel monitoring system, designed for use in non-standard, high-noise, or field applications^21^ and validated in newborns^20^ and age groups relevant to our study^73,74^, was used to collect EEG data. Ground and reference electrodes were Fpz and Oz, respectively. During EEG recordings, electrode impedances were continuously monitored and maintained below 10 kOhms, in accordance with the standards set by the American Clinical Neurophysiology Society^75^. Caregivers assisted by holding or comforting the children during EEG recordings. To keep the children calm and still, caregivers spoke to them softly or read to them while the EEG cap was being applied and during the recording session. The data contained resting-state EEG recordings from 73 children, of whom N = 50 were ZEC (23 female) and N = 23 were UC (11 female).

### Pre-processing

EEG signals recorded at 250 Hz sample rate at the standard 10–20 sites were band pass (0.16–40 Hz) and notch (50 Hz) filtered. The normalized kurtosis of the signals was computed for non-overlapping adjacent time windows of size $\Delta t = 5\;s$. Kurtosis is commonly used for the rejection of ocular and motion artifacts in EEG^76^. A threshold value of 5 for the kurtosis and 1000 μV for the maximum peak-to-peak signal amplitude were selected. Windows exceeding these thresholds were excluded from further analysis (Fig. S3). Recordings were visually inspected by experienced EEG researchers to ensure that the selected threshold values were sufficiently conservative to exclude all artefactual segments. We excluded eight recordings each with a total duration of less than four minutes after pre-processing, leaving 65 recordings for further analysis. To improve space resolution and reduce signal components volume conducted from distant locations we implemented a finite difference Laplace montage^77^. Sensitivity analysis using halved and doubled kurtosis cutoff values yielded minimal impact on the primary developmental patterns observed in the study (Figs. 2, 4; S5–S8).

### EEG feature generation

The frequency band power (FBP) was calculated as the area under the power spectrum within 1 Hz wide frequency bands centred at 1 Hz, 2 Hz, …, 40 Hz. The calculation used ΔW = 5 s Hamming windows, providing sufficient frequency resolution Δf = 1/ΔW = 0.2 Hz for the band power. We calculated the relative FBP to express the band power as a fraction of the total power at the same electrode site. The relative power was preferred as it was expected to be less impacted than absolute power by developmental shifts such as changes in bone thickness and skull resistance^78^ and may have better test–retest reliability^79^. The relative power was calculated in the range 3–30 Hz to minimise the impact from large differences in the absolute power at high or very low frequencies. FBP windows were averaged over the duration of the recording for each participant. We also calculated the Inter-Site Phase-Clustering (ISPC), also known as Phase Locking Value (PLV), for each unordered pair of electrodes within the same frequency bands. ISPC was used to represent the strength of the functional synchrony between pairs of sites^14–16^. In addition, we sought to quantify overall connectivity with a small number of neurobiologically meaningful and easily computable measures with the help of graph theory^49^. For each frequency band an undirected graph was constructed with electrodes representing the nodes and the weight of the edges given by the ISPC. We used weighted functional (rather than binary) connections, as thresholding could eliminate potentially valuable information^48,80^. We next determined the clustering coefficient (CC) and characteristic path length (PL), which are commonly used in network analysis. CC measures how well connected the neighbours of a typical node are to one another, and high values of CC are associated with functional segregation. PL is the mean shortest path over all pairs of nodes, where the distance was calculated from the inverse of ISPC values. PL quantifies the capacity for information transfer across the network, and low values of PL are associated with greater functional integration. We investigated potential developmental trends in FBP, ISPC, CC and PL, by determining the Pearson correlation of the relevant quantity with age, where the correlation was denoted *r*_*N*_ and *r*_*Z*_ for UC and ZEC, respectively.

### Statistical analysis

In assessing group differences, the Wilcoxon Signed-Rank test was used for paired values, e.g., the set of ISPC values in UC for a specific connection versus the set of values in ZEC for the same connection, and the Kolmogorov–Smirnov (KS) test when the groups were unpaired. With age as a covariate, the interaction between Zika status and age was assessed with ANCOVA. To correct for multiple comparisons, the Bonferroni method was applied. Given N = 19 channels, resulting in N(N − 1)/2 = 171 unordered channel pairs, and 40 frequency bands, a total of 171 × 40 = 6840 distinct ISPC values were analysed. The significance threshold was adjusted by dividing the conventional *p*-value of 0.05 by 6840, yielding *p* = 0.00000731 as a criterion for statistical significance to control the family-wise error rate across all comparisons. The above calculations were implemented via custom code in MATLAB and using the functions *signrank*, *mancovan* and *aoctool*.

## Electronic supplementary material

Below is the link to the electronic supplementary material.

Supplementary Material 1

## Data Availability

All data needed to evaluate the conclusions in the paper are present in the paper and/or the Supplementary Materials. All EEG data and analysis scripts are available at 10.5281/zenodo.13311078.
