# Supplementary material for: Disruption of functional network development in children with prenatal Zika virus exposure revealed by resting-state EEG
Source: Sci Rep. 2025 Feb 21;15:6346. doi: 10.1038/s41598-025-90860-0 (PMC11845516; doi:10.1038/s41598-025-90860-0)
Supplement: Supplementary file 1 — Supplementary Material 1 [file 41598_2025_90860_MOESM1_ESM.docx]

Supplementary Materials for

**Disruption of functional network development in children with prenatal Zika virus exposure revealed by resting-state EEG**

Ahmet Omurtag* *et al.*

* Corresponding author. Email: ahmet.omurtag@ntu.ac.uk

**Participants and data collection.** Institutional Review Board approval was obtained at St George’s University (IRB#16061) and Stanford University (IRB#45242). Informed consent was obtained from all mothers who participated in this study. There was no financial compensation. Mother-infant pairs were prospectively enrolled between April 2016 and March 2017. Maternal serum samples were initially assessed for flavivirus exposure with indirect IgG capture Enzyme-Linked Immunosorbent Assay (ELISA) using dengue virus (DENV1-4) antigen ^81^ and a multiplexed assay on a nanostructured plasmonic gold (pGOLD) platform (Nirmidas Biotech, Palo Alto, CA) for the detection of IgG and IgG avidity against ZIKV and DENV antigens. The pGOLD IgG immunoassay was used to cross-validate ELISA results and distinguish ZIKV from DENV antigens, as it has demonstrated sensitivity and specificity to ZIKV greater than 90% and 98%, respectively, in the convalescent phase ^82^. IgG avidity testing provided further data on the timing of ZIKV and DENV infection. One hundred and fourteen children were classified as ZIKV-Exposed Children (ZEC) because they were born to mothers who were ZIKV-Infected during pregnancy. One hundred and twenty 120 children were classified as Unexposed Children (UC) because they were born to mothers who tested negative for ZIKV during the prenatal and postnatal period. EEG recordings from ZIKV exposed children (ZEC, N=50 with 23 female) and unexposed children (UC, N=23 with 11 female) were obtained, totaling 73 recordings (34 female).

**ZIKV Testing and Sociodemographic Assessment.** Maternal and child serum samples were assessed for ZIKV exposure with indirect IgG capture Enzyme-Linked Immunosorbent Assay (ELISA) and the pGOLD IgG immunoassay. There were no differences in age, head circumference, weight, height, or household food security between the children. A structured interview and questionnaire set to primary caregivers to determine whether ZEC and UC were similar in terms of household socio-demographics, such as maternal age at delivery and food security was administered ^83^. Child anthropometrics (head circumference, weight, and height) were assessed to determine whether ZEC were comparable to UC in physical growth at the time of outcome assessment, using World Health Organization and Center for Disease Control Growth Standards ^84^. Sociodemographic comparisons between the groups were done via structured questionnaires set to primary caregivers and child anthropometrics were assessed to determine comparable physical growth using World Health Organization and Center for Disease Control Growth Standards. Results can be found in Table S1. Handedness of the children or their parents was unknown, the children were too young. The children in the final analyses were selected from a subset of the children in Table S1 below, based on the need to equate age bands across the ZEC and UC groups.

Table S1. Demographic Characteristics of the Sample.

|  | ZEC (N=50) | UC (N=23) |
| --- | --- | --- |
| Male | 27 (54%) | 12 (52%) |
| Female | 23 (46%) | 11 (48%) |
|  | **Mean (SD)** | |
| Mother’s Age at Delivery (years) | 28.77 (5.62) | 28.49 (7.62) |
| Gestational Age at Delivery (weeks) | 39.80 (1.47) | 38.89 (1.71) |
| Child’s Age (in months) | 22.15 (4.10) | 24.68 (2.40) |
| Child’s Head Circumference (z-score*) | 0.49 (0.91) | 0.32 (1.03) |
| Child’s Weight (z-score*) | 0.41 (1.18) | 0.16 (1.47) |
| Child’s Height (z-score*) | 0.35 (1.02) | 0.18 (1.01) |
| *Z-scores were calculated using World Health Organization Child Growth Standards. | | |

**Neurodevelopmental Assessment.** Cognition, motor skills, language skills, and behavior were clinically assessed with the INTER-NDA ^72^. The standardized scores were ≥38.5 for cognition, ≥25.7 for fine motor, ≥51.7 for gross motor, and ≥17.8 for language. The Cardiff Acuity Test (CAT) ^85^ was used to assess visual acuity, which is the ability to detect fine visual detail. Resulting “Logarithm of the Minimum Angle of Resolution (LogMAR)” scores were used for analyses, they range from 0.1 to 1.0, with lower scores indicating better visual acuity ^86,87^. The CAT has been validated for use in toddlers and normative scores for children between 24 and 29.9 months of age range from 0.1 to 0.418. The Cardiff Contrast Test (CCT) was used to assess contrast sensitivity, which is the ability to visually detect large but faint objects ^87^. Administration was like the CAT with vanishing optotypes that progressively decrease in light/dark contrast. Normative estimates are available for children between 24 to 36 months of age and range from 33.3 to 100, with higher scores indicating better contrast sensitivity.

Table S2 displays the demographics and test scores for the participants whose EEGs met the inclusion criteria for signal quality (see main article’s Methods section).

Table S2. Participant demographics and test scores.

| Subject number | Age  (days) | Age  (mo) | Recording  duration (s) | Zika status | VisionLogMAR | VisionCS | MeanCog | MeanFineMotor | MeanGrossMotor | MeanReceptLang | MeanExpressLang | MeanLanguage | MeanOverall |
| --- | --- | --- | --- | --- | --- | --- | --- | --- | --- | --- | --- | --- | --- |
| 1 | 790 | 25.97 | 625 | 1 | 0.2 | 0 | 2.67 | 4 | 4 | 3 | 3.78 | 3.73 | 3.38 |
| 2 | 731 | 24.03 | 385 | 1 | 0 | 0 | 3.1 | 4 | 3.33 | 4 | 3.44 | 3.5 | 3.3 |
| 3 | 734 | 24.13 | 750 | 1 | 0 | 0 | 2.69 | 4 | 3.33 | 4 | 3.3 | 3.25 | 3.27 |
| 4 | 759 | 24.95 | 845 | 1 | 0.4 | 66.66 | 2.77 | 4 | 3.33 | 4 | 3.1 | 2.92 | 3.27 |
| 5 | 724 | 23.8 | 870 | 1 | 0.2 | 50 | 3.46 | 4 | 3.67 | 4 | 3.6 | 3.58 | 3.63 |
| 6 | 745 | 24.49 | 400 | 0 | 0 | 0 | 2 | 3.25 | 4 | 2 | 2.6 | 2.42 | 2.62 |
| 7 | 730 | 24 | 780 | 1 | 0 | 0 | 2.33 | 4 | 3.67 | 4 | 2.3 | 2.18 | 2.71 |
| 8 | 735 | 24.16 | 820 | 1 | 0 | 0 | 3.15 | 3.25 | 3.33 | 4 | 3.5 | 3.5 | 3.33 |
| 9 | 488 | 16.04 | 910 | 1 | 0 | 0 | 2.75 | 4 | 4 | 3.5 | 3.56 | 3.27 | 3.45 |
| 10 | 719 | 23.64 | 1150 | 1 | 0.6 | 16.67 | 2.38 | 3.5 | 4 | 3 | 3 | 2.75 | 3 |
| 11 | 663 | 21.8 | 1050 | 1 | 0 | 0 | 0 | 0 | 0 | 0 | 0 | 0 | 0 |
| 12 | 785 | 25.81 | 300 | 0 | 0 | 0 | 2.82 | 4 | 4 | 3 | 1.67 | 1.9 | 2.71 |
| 13 | 720 | 23.67 | 785 | 0 | 0 | 0 | 3.38 | 4 | 4 | 4 | 3.3 | 3.42 | 3.47 |
| 14 | 913 | 30.02 | 710 | 0 | 0 | 0 | 0 | 0 | 0 | 0 | 0 | 0 | 0 |
| 15 | 743 | 24.43 | 785 | 0 | 0.1 | 50 | 2.92 | 4 | 3 | 4 | 1.7 | 2 | 2.93 |
| 16 | 609 | 20.02 | 825 | 1 | 0 | 0 | 3.55 | 3.75 | 4 | 4 | 3 | 3.18 | 3.39 |
| 17 | 741 | 24.36 | 890 | 1 | 0.4 | 33.33 | 2.69 | 3.67 | 3.67 | 2 | 3.38 | 3.5 | 3.22 |
| 18 | 752 | 24.72 | 470 | 1 | 0.2 | 25 | 3.31 | 4 | 4 | 3.5 | 3.1 | 3.25 | 3.57 |
| 19 | 739 | 24.3 | 695 | 1 | 0 | 0 | 2.54 | 4 | 4 | 4 | 2.11 | 2.18 | 2.86 |
| 20 | 743 | 24.43 | 1340 | 0 | 0.1 | 0 | 2.38 | 3.25 | 4 | 3.5 | 2.8 | 2.5 | 3 |
| 21 | 755 | 24.82 | 685 | 1 | 0 | 0 | 2.83 | 3.75 | 3.33 | 4 | 2.2 | 2.5 | 2.96 |
| 22 | 755 | 24.82 | 1425 | 1 | 0.4 | 33.33 | 2.62 | 3.33 | 4 | 3 | 1.6 | 1.5 | 2.48 |
| 23 | 741 | 24.36 | 485 | 1 | 0.6 | 16.67 | 2.73 | 4 | 4 | 3.5 | 3.4 | 3.45 | 3.37 |
| 24 | 733 | 24.1 | 695 | 1 | 0.3 | 0 | 2.92 | 4 | 4 | 4 | 3.4 | 3.42 | 3.45 |
| 25 | 734 | 24.13 | 950 | 0 | 0.5 | 0 | 2.62 | 3.33 | 3.33 | 4 | 2.7 | 2.75 | 2.79 |
| 26 | 527 | 17.33 | 1295 | 1 | 0 | 0 | 3.54 | 4 | 3.67 | 4 | 3.7 | 3.75 | 3.7 |
| 27 | 912 | 29.98 | 400 | 0 | 0.1 | 0 | 2.36 | 4 | 3.33 | 2.5 | 2.7 | 2.64 | 2.79 |
| 28 | 368 | 12.1 | 300 | 1 | 0.5 | 16.67 | 2.54 | 3.25 | 3 | 3 | 2.1 | 2.33 | 2.6 |
| 29 | 384 | 12.62 | 1325 | 1 | 0 | 0 | 0 | 0 | 0 | 0 | 0 | 0 | 0 |
| 30 | 749 | 24.62 | 330 | 1 | 0.2 | 66.66 | 2.83 | 3.75 | 4 | 4 | 2.67 | 2.91 | 3.04 |
| 31 | 422 | 13.87 | 1900 | 1 | 0 | 0 | 0 | 0 | 0 | 0 | 0 | 0 | 0 |
| 32 | 913 | 30.02 | 770 | 1 | 0.4 | 50 | 3.38 | 4 | 4 | 4 | 3 | 3.17 | 3.47 |
| 33 | 578 | 19 | 510 | 0 | 0 | 0 | 3.46 | 4 | 4 | 4 | 3.5 | 3.58 | 3.73 |
| 34 | 847 | 27.85 | 865 | 1 | 0 | 0 | 3.23 | 3 | 3.67 | 4 | 3 | 2.92 | 3.27 |
| 35 | 733 | 24.1 | 1005 | 0 | 0 | 0 | 3 | 3.75 | 4 | 3.5 | 2.9 | 3 | 3.2 |
| 36 | 713 | 23.44 | 330 | 1 | 0 | 0 | 3.08 | 4 | 3.33 | 4 | 2.8 | 3 | 3.17 |
| 37 | 731 | 24.03 | 410 | 1 | 0 | 0 | 3.75 | 4 | 4 | 3.78 | 4 | 3.82 | 3.83 |
| 38 | 745 | 24.49 | 965 | 1 | 0 | 0 | 2.25 | 4 | 4 | 0 | 1.75 | 1.75 | 2.53 |
| 39 | 761 | 25.0 | 835 | 1 | 0 | 0 | 3.46 | 4 | 3.33 | 4 | 3.8 | 3.58 | 3.67 |
| 40 | 720 | 23.7 | 465 | 1 | 0 | 0 | 3.56 | 4 | 4 | 4 | 2.11 | 2.3 | 3.12 |
| 41 | 516 | 17.0 | 1065 | 1 | 0 | 0 | 0 | 0 | 0 | 0 | 0 | 0 | 0 |
| 42 | 425 | 14.0 | 1415 | 1 | 0 | 0 | 2.83 | 4 | 4 | 4 | 2.38 | 2.1 | 2.93 |
| 43 | 734 | 24.1 | 380 | 0 | 0.3 | 0 | 3.38 | 4 | 4 | 4 | 2.7 | 2.92 | 3.4 |
| 44 | 726 | 23.9 | 730 | 1 | 0.6 | 33.33 | 3.15 | 3.25 | 3 | 4 | 2.8 | 3 | 3.2 |
| 45 | 670 | 22.0 | 410 | 1 | 0 | 0 | 2.69 | 4 | 4 | 4 | 2.2 | 2.08 | 2.73 |
| 46 | 734 | 24.1 | 660 | 1 | 0 | 0 | 3.15 | 4 | 4 | 3.5 | 3.4 | 3.42 | 3.53 |
| 47 | 734 | 24.1 | 1395 | 0 | 0.1 | 66.66 | 3.58 | 4 | 3.33 | 4 | 3.4 | 3.45 | 3.71 |
| 48 | 743 | 24.4 | 640 | 0 | 0 | 0 | 3 | 4 | 3.33 | 3 | 3.5 | 3.33 | 3.38 |
| 49 | 730 | 24.0 | 1005 | 1 | 0 | 0 | 2.58 | 4 | 4 | 2.5 | 3 | 2.9 | 3 |
| 50 | 732 | 24.1 | 910 | 1 | 0.5 | 0 | 2.82 | 4 | 4 | 4 | 3.56 | 3.45 | 3.39 |
| 51 | 749 | 24.6 | 495 | 1 | 0.5 | 16.67 | 2.67 | 3.5 | 3.67 | 4 | 3.1 | 3.25 | 3.17 |
| 52 | 833 | 27.4 | 1040 | 1 | 0.5 | 0 | 1.92 | 3.25 | 3.67 | 1.5 | 3.2 | 3.27 | 2.83 |
| 53 | 736 | 24.2 | 545 | 0 | 0.1 | 66.66 | 2.58 | 4 | 3.33 | 4 | 1.6 | 1.64 | 2.55 |
| 54 | 732 | 24.1 | 890 | 1 | 0.4 | 0 | 2.92 | 4 | 4 | 3.5 | 3.44 | 3.45 | 3.31 |
| 55 | 824 | 27.1 | 1045 | 1 | 0.2 | 33.33 | 3.5 | 3.67 | 4 | 4 | 2.67 | 2.75 | 3.5 |
| 56 | 722 | 23.7 | 515 | 1 | 0 | 0 | 2.7 | 4 | 4 | 2.5 | 1.75 | 2.1 | 2.65 |
| 57 | 754 | 24.8 | 250 | 0 | 0.1 | 50 | 3.33 | 3.75 | 4 | 4 | 3.56 | 3.6 | 3.6 |
| 58 | 715 | 23.5 | 510 | 1 | 0 | 0 | 1.92 | 4 | 3.33 | 3.5 | 1.6 | 1.55 | 2.31 |
| 59 | 778 | 25.6 | 305 | 1 | 0 | 0 | 3.17 | 4 | 4 | 3 | 3.5 | 3.58 | 3.59 |
| 60 | 741 | 24.4 | 915 | 1 | 0.3 | 66.66 | 2.69 | 3 | 3.33 | 3.5 | 2.89 | 2.73 | 2.83 |
| 61 | 718 | 23.6 | 590 | 1 | 0 | 0 | 3.75 | 3.67 | 4 | 4 | 3.33 | 3.45 | 3.54 |
| 62 | 734 | 24.1 | 945 | 0 | 0.4 | 33.33 | 3.5 | 3.75 | 4 | 4 | 3.67 | 3.45 | 3.43 |
| 63 | 760 | 25.0 | 325 | 0 | 0 | 0 | 0 | 0 | 0 | 0 | 0 | 0 | 0 |
| 64 | 743 | 24.4 | 975 | 0 | 0.4 | 50 | 3.33 | 3.25 | 4 | 4 | 3.2 | 3.27 | 3.45 |
| 65 | 790 | 26.0 | 485 | 0 | 0.4 | 33.33 | 3.08 | 3.5 | 3.33 | 4 | 2 | 2.2 | 2.89 |


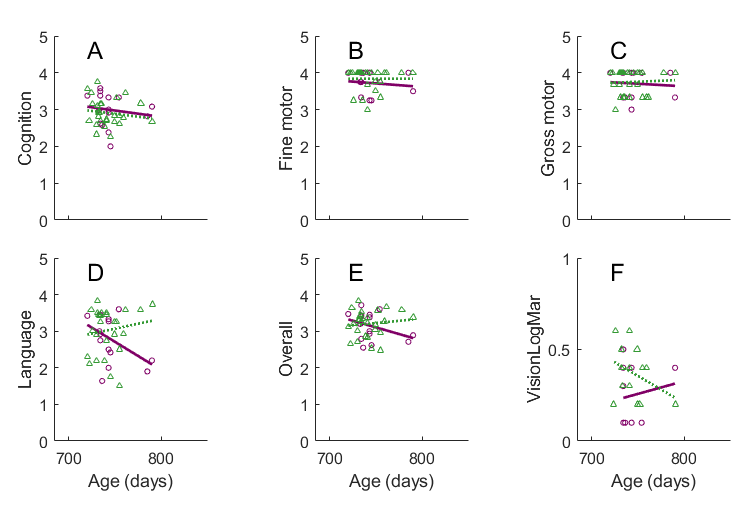


**Fig. S1. Test scores and age.** Blue and red dots indicate normal and zika exposed subjects while the blue and red lines are the corresponding regression lines fitted to the data points. None of the group differences reached statistical significance. The medians for UC and ZEC and the p-value of their difference are as follows: (**A**) Cognition (-0.11, -0.11, 0.89); (**B**) Fine motor (0.11, -0.29, 0.47); (**C**) Gross motor (-0.11, 0.10, 0.77); (**D**) Language (-0.14, 0, 0.61); (**E**) Overall (-0.11, 0.01, 0.69); (**F**) Vision LogMar (0.51, -0.18, 0.06).


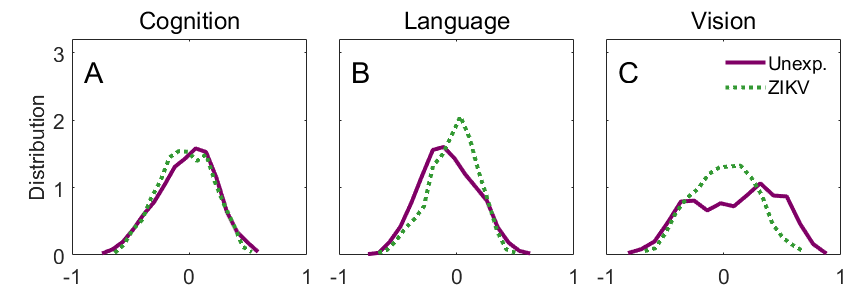


**Fig. S2. Histograms of correlations between neurodevelopmental test scores and age.** Unexposed (solid) and ZIKV exposed children (dotted) for (**A**) Cognition; (**B**) Language; and (**C**) Vision LogMar.


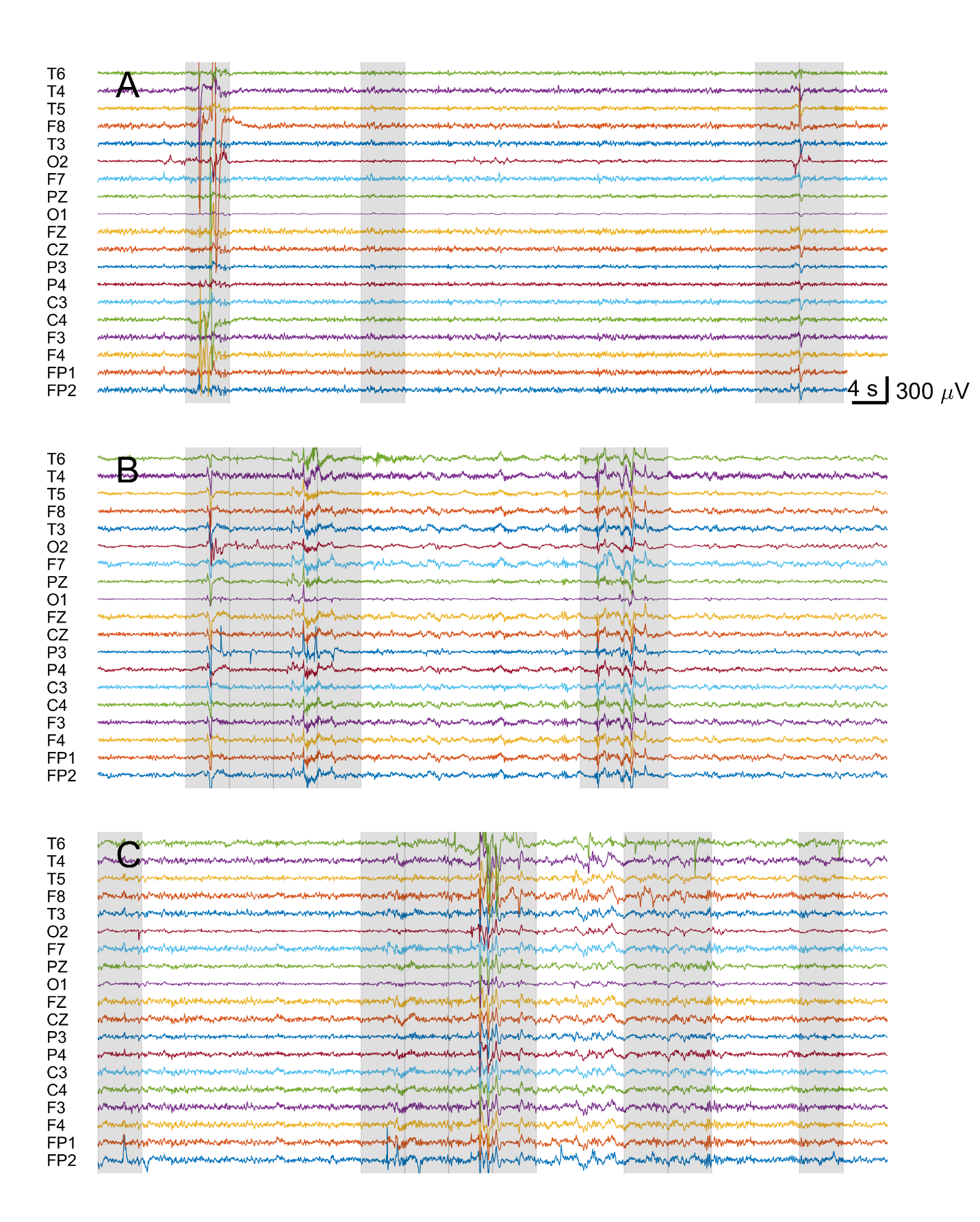


**Fig. S3. Examples of EEG artifact minimisation.** The EEG segment in the figure shows intervals (gray shaded) with artifacts, which were automatically detected and excluded from further analysis. (A) Participant ID 74; (B) 34; (C) 30.


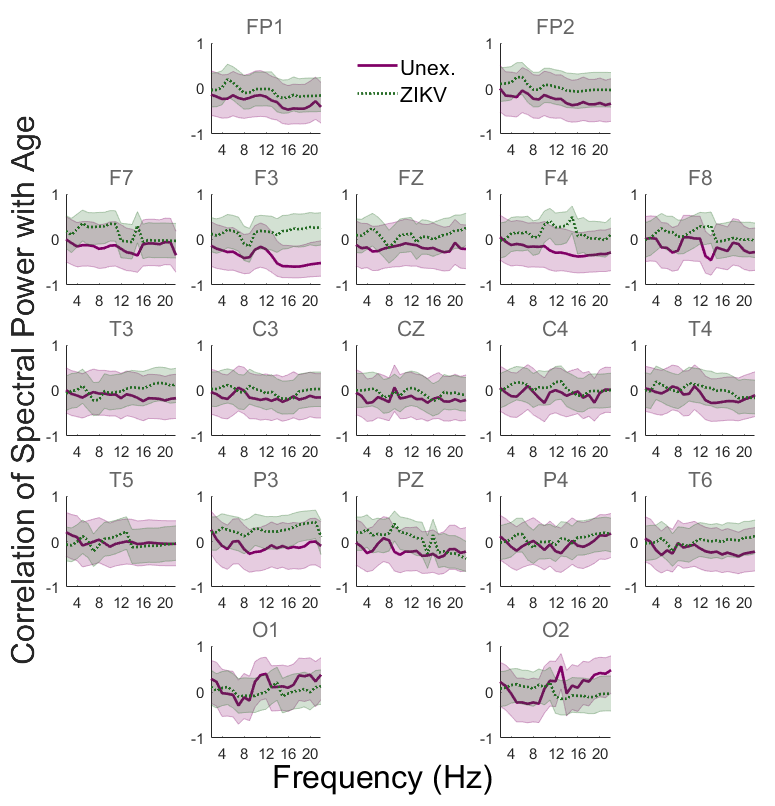


**Fig. S4 The correlation between relative frequency band power and age as a function of the frequency.** Unexposed (solid) and ZIKV exposed (dotted) children are shown with shaded regions indicating the sample standard error across the corresponding group of participants.


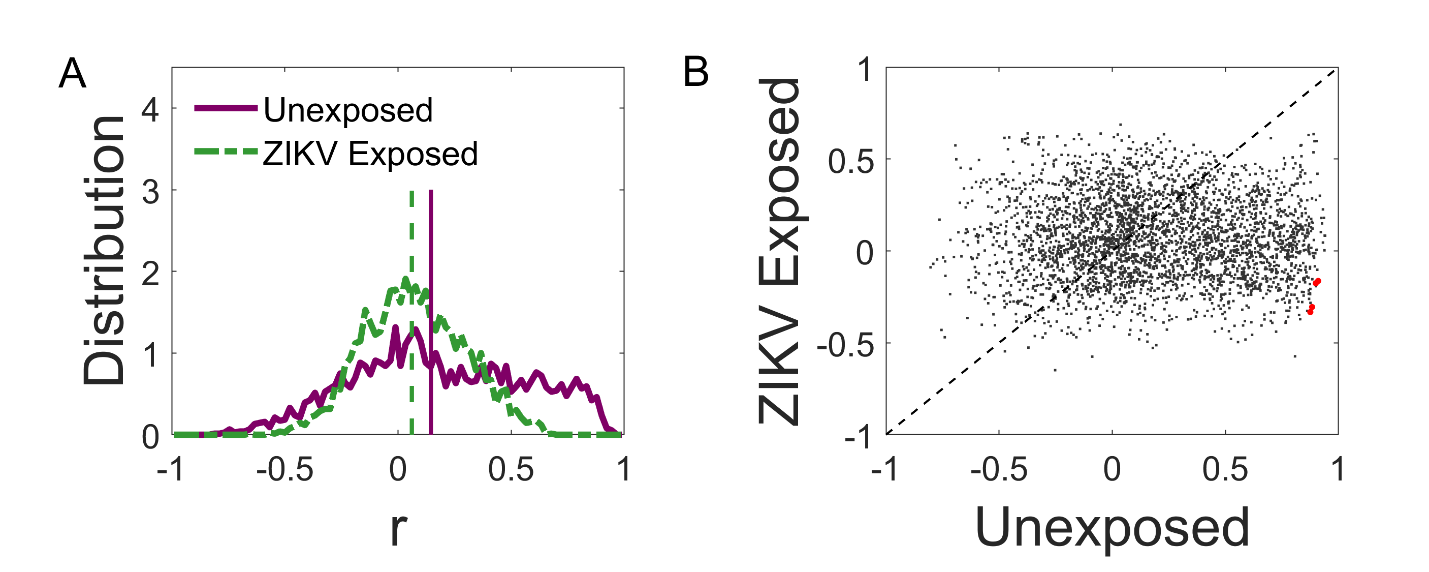


**Fig. S5** Same as Fig. 2 in the main text, except the ISPCs were calculated using a cutoff value of 2.5 for the normalized kurtosis in selecting valid recordings during preprocessing.


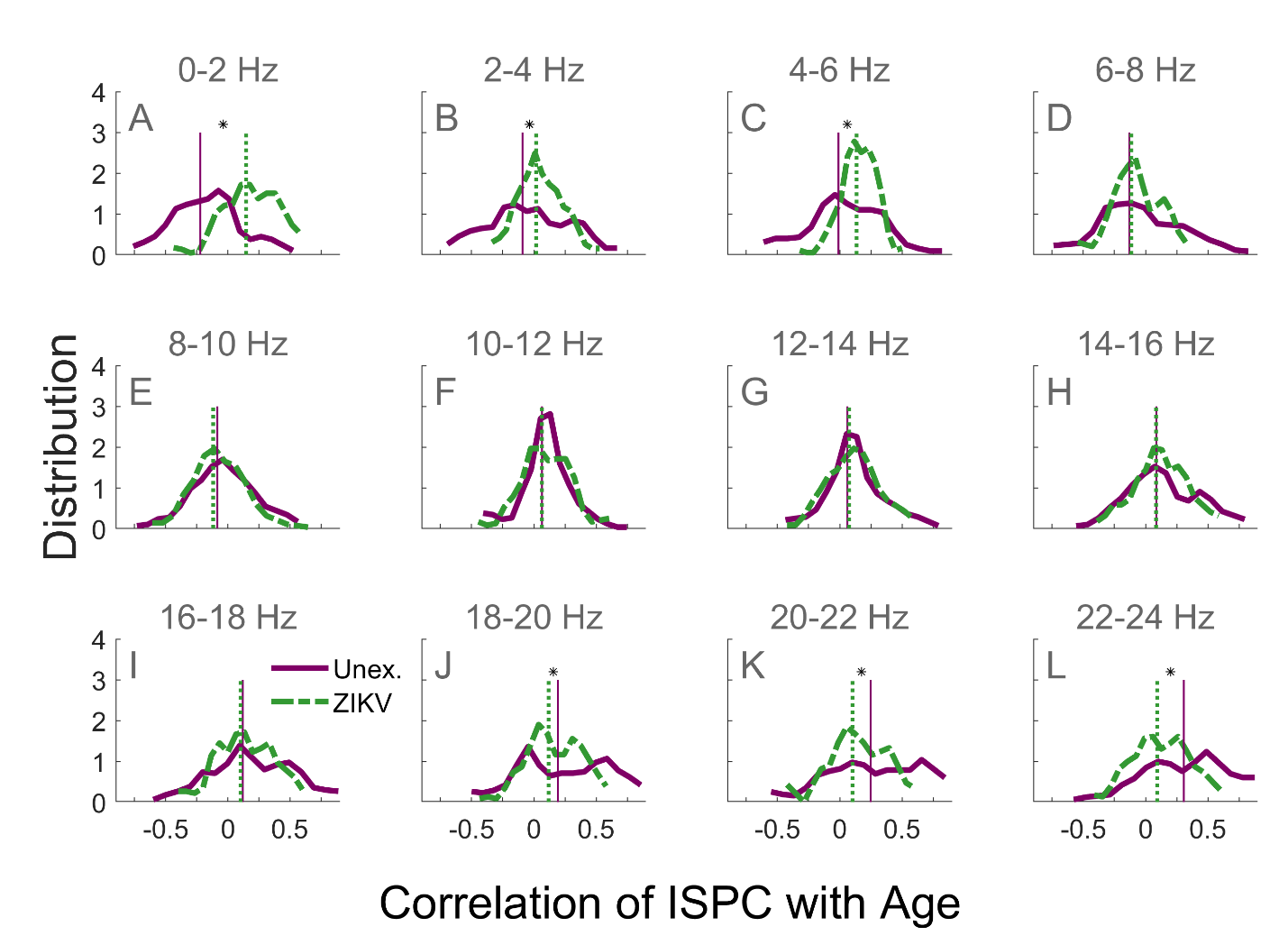


**Fig. S6** Same as Fig. 4 in the main text, except the ISPCs were calculated using a cutoff value of 2.5 for the normalized kurtosis in selecting valid recordings during preprocessing.


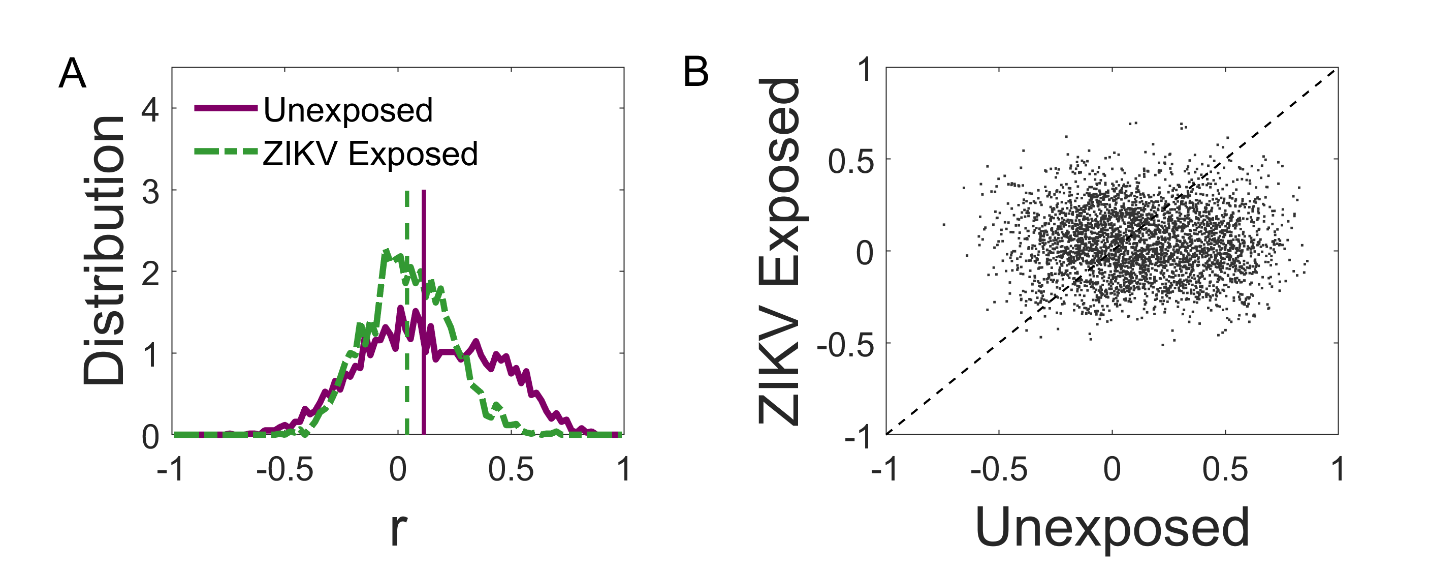


**Fig. S7** Same as Fig. 2 in the main text, except the ISPCs were calculated using a cutoff value of 10 for the normalized kurtosis in selecting valid recordings during preprocessing.


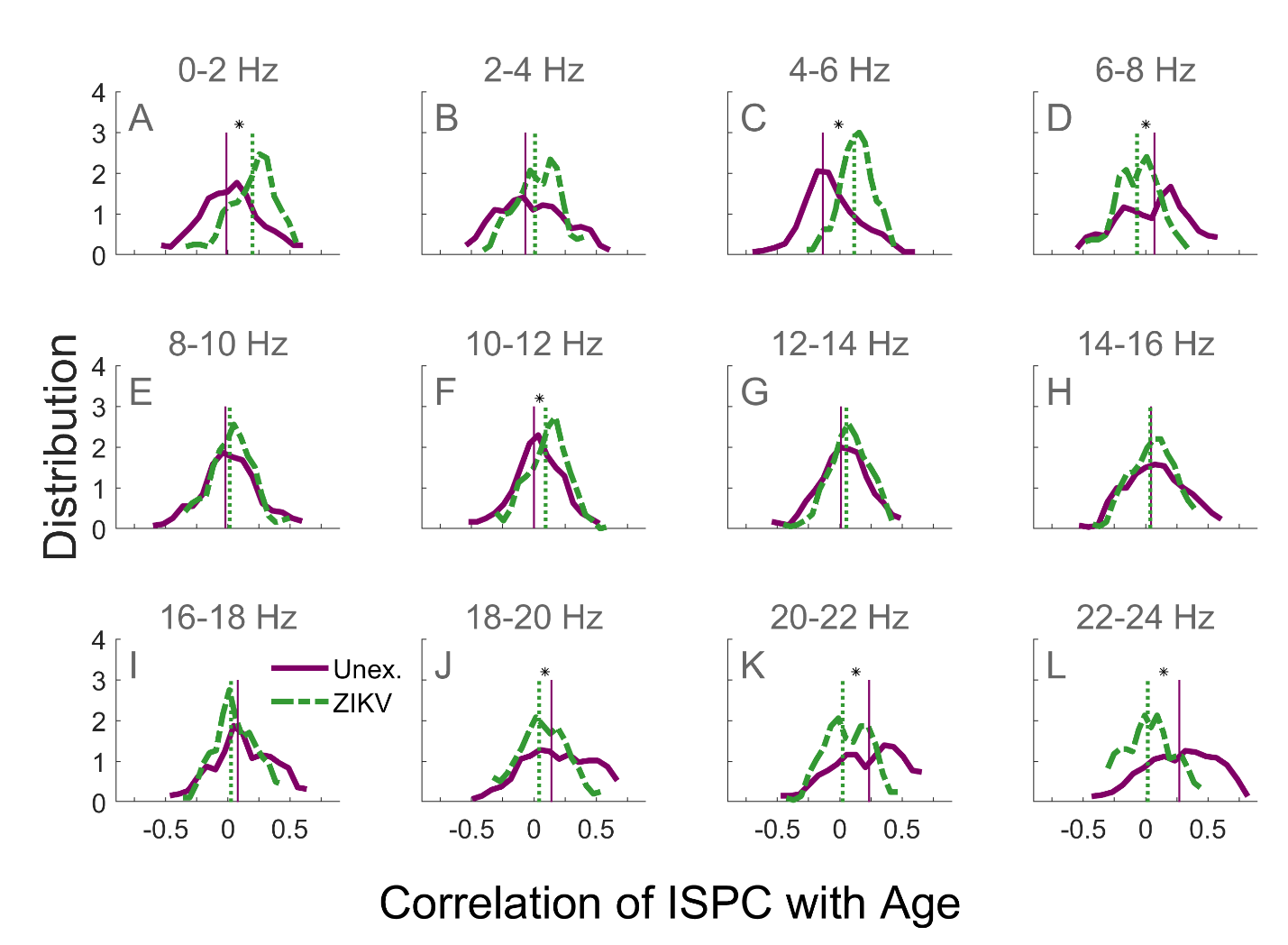


**Fig. S8** Same as Fig. 4 in the main text, except the ISPCs were calculated using a cutoff value of 10 for the normalized kurtosis in selecting valid recordings during preprocessing.

REFERENCES

81. Grossi-Soyster, E. N. *et al.* Serological and spatial analysis of alphavirus and flavivirus prevalence and risk factors in a rural community in western Kenya. *PLoS Negl Trop Dis* **11**, (2017).

82. Zhang, B. *et al.* Diagnosis of Zika virus infection on a nanotechnology platform. *Nat Med* **23**, (2017).

83. Bickel, G., M, N., C, P., W, H. & Cook, J. Guide to Measuring Household Food Security. USDA Food and Nutrition Service. *Office of Analyses, Nutrition, and Evaluation* (2000).

84. Baker, R. D. *et al.* Clinical report - Diagnosis and prevention of iron deficiency and iron-deficiency anemia in infants and young children (0-3 years of age). *Pediatrics* **126**, (2010).

85. Adoh, T. O. & Woodhouse, J. M. The Cardiff acuity test used for measuring visual acuity development in toddlers. *Vision Res* **34**, 555–560 (1994).

86. Rosser, D. A., Laidlaw, D. A. H. & Murdoch, I. E. The development of a ‘reduced logMAR’ visual acuity chart for use in routine clinical practice. *British Journal of Ophthalmology* **85**, (2001).

87. Barbareza, R., Woodhouse, M. & Oduwaiye, K. A new contrast sensitivity test for young children–The Cardiff Contrast Test. *Ophthalmic and Physiological Optics* **17**, 175 (1997).
